# The Trim32-DPEP2 axis is an inflammatory switch in macrophages during intestinal inflammation

**DOI:** 10.1038/s41418-025-01468-w

**Published:** 2025-02-28

**Authors:** Zhiyan Zhan, Huisheng Liang, Zhuoqi Zhao, Liya Pan, Jing Li, Yuyun Chen, Zhoulonglong Xie, Zhilong Yan, Ying Xiang, Wenxue Liu, Li Hong

**Affiliations:** 1 Department of Clinical Nutrition, Shanghai Children’s Medical Center, Shanghai Jiao Tong University School of Medicine, Shanghai, 200127 China; 2 Clinical Research Center, Shanghai Children’s Medical Center, Shanghai Jiao Tong University School of Medicine, Shanghai, 200127 China; 3 Department of Obstetrics and Gynecology, Zhongshan Hospital, Fudan University, Shanghai, China; 4 Department of Gynecology, Zhongshan Hospital (Xiamen), Fudan University, Xiamen, 361000 China; 5 Fujian Children’s Hospital (Fujian Branch of Shanghai Children’s Medical Center), College of Clinical Medicine for Obstetrics & Gynecology and Pediatrics, Fujian Medical University, Fuzhou, China; 6 Department of Surgery, Shanghai Children’s Medical Center, Shanghai Jiao Tong University School of Medicine, Shanghai, 200127 China; 7 Department of Laboratory Medicine, Shanghai Children’s Medical Center, Shanghai Jiao Tong University School of Medicine, Shanghai, 200127 China

**Keywords:** Ubiquitin ligases, Immunological disorders

## Abstract

The mechanisms via which inflammatory macrophages mediate intestinal inflammation are not completely understood. Herein, using merged analysis of RNA sequencing and mass spectrometry-based quantitative proteomics, we detected differences between proteomic and transcriptomic data in activated macrophages. Dipeptidase-2 (DPEP2), a member of the DPEP family, was highly expressed and then downregulated sharply at the protein level but not at the mRNA level in macrophages in response to inflammatory stimulation. Suppression of DPEP2 not only enhanced macrophage-mediated intestinal inflammation in vivo but also promoted the transduction of inflammatory pathways in macrophages in vitro. Mechanistically, overexpressed DPEP2 inhibited the transduction of inflammatory signals by resisting MAK3K7 in inactivated macrophages, whereas DPEP2 degradation by activated Trim32 resulted in strong activation of NF-κB and p38 MAPK signaling via the release of MAK3K7 in proinflammatory macrophages during the development of intestinal inflammation. The Trim32-DPEP2 axis accumulates the potential energy of inflammation in macrophages. These results identify DPEP2 as a key regulator of macrophage-mediated intestinal inflammation. Thus, the Trim32-DPEP2 axis may be a potential therapeutic target for the treatment of intestinal inflammation.

## Introduction

Intestinal inflammation severely affects the nutritional status and normal development of children. Our previous study revealed that the LPS released from *Veillonella parvula* (LPS-V) promoted the development of Hirschsprung’s enterocolitis (HAEC)-like intestinal inflammation by inducing macrophage dysfunction [1]. In addition, multiple studies have addressed the important role of macrophages in promoting the development of various types of intestinal inflammation, including inflammatory bowel disease (IBD) [2], necrotizing enterocolitis [3], and HAEC [4, 5]. Various mechanisms are involved in the regulation of inflammatory signal transduction during the activation of macrophages, which is valuable for developing targeted therapy strategies.

It is well known that macrophages emerge as immune guardians of the healthy organism [6, 7]. However, macrophage dysfunction often acts as a key factor that impairs recovery from inflammatory diseases [1, 8]. The transduction of inflammatory signals in macrophages is very complex, and various key molecules, including TLR4, NF-κB, p38, and Erk1/2, participate in this process [9–12]. Investigating the mechanisms by which inflammatory signals are dysregulated in macrophages is essential and provides a theoretical basis for novel targeted therapeutic strategies.

In response to inflammatory stimulation, inflammatory signals are strongly initiated and transduced in macrophages [13, 14]. In addition, the proinflammatory activation of macrophages from the inactivated state, also known as M1 polarization, can be completed in an extremely short time [15–17], indicating the potential energy of inflammation in inactivated macrophages. Although multiple previous studies have demonstrated the transduction of inflammatory signals in macrophages, the mechanisms by which inflammatory signals are strongly transduced remain obscure.

The dipeptidase (DPEP) family, which comprises DPEP1, DPEP2, and DPEP3, plays an essential role in the development of inflammation [18–21]. Both DPEP1 and DPEP2 promote the conversion of leukotriene D4 to leukotriene E4 [22], and DPEP2 is overexpressed in some inflammatory diseases [20, 21, 23], which suggests the potential effects of DPEP2 on inflammation. However, the mechanisms by which DPEP2 regulates inflammation remain unknown.

Tripartite motif-containing protein 32 (Trim32), which acts as an E3 ubiquitin-protein ligase, is essential for multiple immune processes, including antiviral responses, innate immune responses, and inflammatory skin disorders [24–27]. Moreover, Trim32 is involved in various molecular pathways related to inflammation, such as type I interferon signaling, NF-κB or STING signaling, and Piasy function [26, 28–30]. Trim32 is activated through self-ubiquitination and functions in the macrophage response to inflammatory stimulation.

In this study, we investigated the proteomic and transcriptomic alterations in LPS-V-activated macrophages. The DPEP2 protein was identified as an essential regulator of inflammatory signal transduction by MAP3K7 in macrophages. The Trim32-DPEP2 axis accumulates the potential energy of inflammation in macrophages and is a potential therapeutic target for the treatment of macrophage-mediated intestinal inflammation.

## Results

### Proteomic and transcriptomic analyses of activated macrophages

**Fig. 1:**
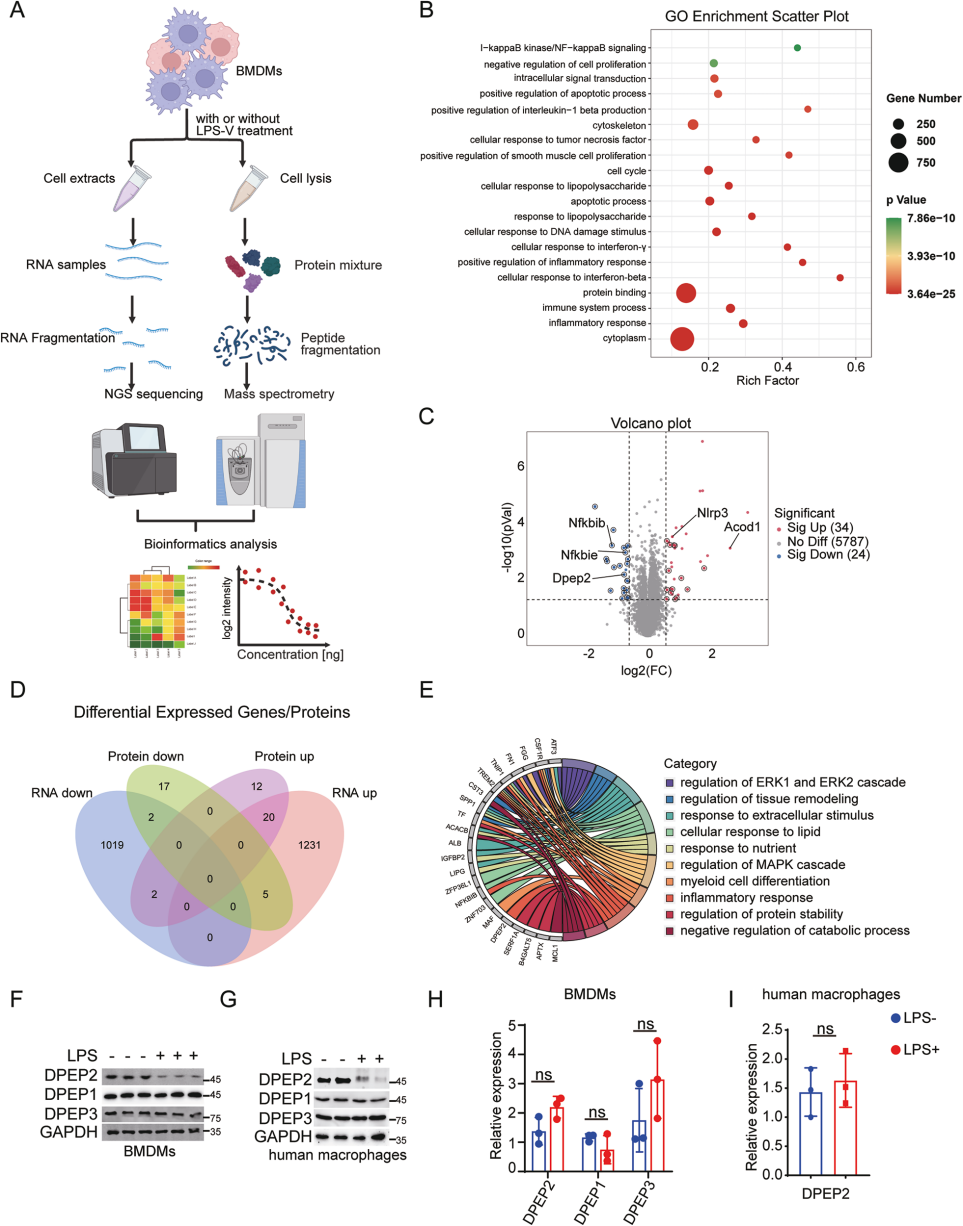
Analysis of proteomics and transcriptomics in activated macrophages. **A** Pattern diagram of proteomics and transcriptomics analysis in macrophages with or without inflammatory stimulation. **B** GO functional enrichment analysis of differentially expressed genes (DEGs) of transcriptomics in BMDMs with or without LPS-V treatment (1 μg/ml, 2 h). **C** Volcano map showing proteomic differentially expressed genes in BMDMs with or without LPS-V treatment (1 μg/ml, 2 h). Red indicates highly expressed proteins by LPS-V; Blue indicates low expression proteins by LPS-V; The circles represent proteins that were inconsistent with RNA sequencing; Key proteins are labeled by the protein names. **D** Venn diagram shows the number of identical or different genes for differentially expressed genes as determined by RNA sequencing and quantitative proteomics. RNA down, RNA up, Protein down and Protein up represent low expression in RNA seq, high expression in RNA seq, low expression in proteome, and high expression in proteome induced by LPS-V, respectively. **E** GO functional enrichment analysis of the differentially expressed proteins whose correspondent mRNA levels don’t show the same alteration. LPS treatments decrease the protein expression of DPEP2 but not DPEP1 or DPEP3 in BMDMs (**F**) and human macrophages (**G**). LPS treatments have no significant influence on the mRNA expression of DPEP1/2/3 in BMDMs (**H**) and human macrophages (**I**). All data are expressed as mean ± SD. ns denotes no signification.

To further investigate the mechanism by which macrophages are activated by LPS-V [1], we employed RNA sequencing and mass spectrometry (MS)-based quantitative (4D-DIA) proteomics to identify key regulators of inflammatory signal transduction in LPS-V-stimulated macrophages (Fig. 1A) [31, 32]. Briefly, bone marrow-derived macrophages (BMDMs) were collected after 2 h of treatment with LPS-V, then lysed and subjected to RNA sequencing or MS-based quantitative proteomics. RNA sequencing revealed that 1256 genes were upregulated and 1023 genes were downregulated in macrophages by LPS-V treatment (Fig. S1A, B and Table S1). GO analysis revealed that the LPS-V-induced DEGs were involved in multiple classical inflammatory pathways (Fig. 1B). Moreover, transcriptome similarity was observed between LPS-V-treated and LPS from *E. coli* (LPS-E)-treated macrophages (Fig. S1A–D and Table S2). On the other hand, 34 proteins whose expression was upregulated and 24 proteins whose expression was downregulated in macrophages in response to LPS-V treatment were identified by MS-based quantitative proteomics analysis (Fig. 1C and Table S3). Among these differentially expressed proteins, multiple inflammation-related proteins, including NLRP3, ACOD1, NFKBIB, and NFKBIE, were identified, which have also been reported previously [33–36] (Fig. 1D, E). In addition, LPS-V stimulation induced the differential expression of several proteins, such as Lipg, Wbp4 and Tmco3, which have not been reported to be closely related to inflammation (Table S3). Strikingly, merged analysis revealed low consistency between the transcriptomic and proteomic data (Fig. 1C, D and Table S4).

We next investigated the distinctions between the proteomic and transcriptomic data in activated macrophages. Owing to the lower sensitivity of MS than that of RNA sequencing, some differentially expressed proteins were undetectable by 4D-DIA proteomics, whereas the corresponding DEGs could be identified. To avoid false-negative results for differentially expressed proteins by MS analysis, only differentially expressed proteins whose corresponding mRNA levels did not show the same alteration were further analyzed. Following this strategy, 36 proteins were identified, among which DPEP2 protein expression was downregulated in activated macrophages (Fig. 1D and Table S4). In contrast, previous reports have shown that the DPEP family, including DPEP1, DPEP2, and DPEP3, plays an essential role in the development of inflammation [18–21]. Interestingly, the DPEP2 protein, but not the DPEP1 or DPEP3 protein, was downregulated in LPS-treated macrophages (Fig. 1F, G), whereas the mRNA levels of all the 3 DPEPs did not vary significantly (Fig. 1H, I).

Taken together, comparison between proteomic and transcriptomic data in activated macrophages revealed that the DPEP2 protein but not mRNA was downregulated, and thus more research is needed to validate the role of DPEP2 in macrophages.

### DPEP2 is upregulated in macrophages

**Fig. 2:**
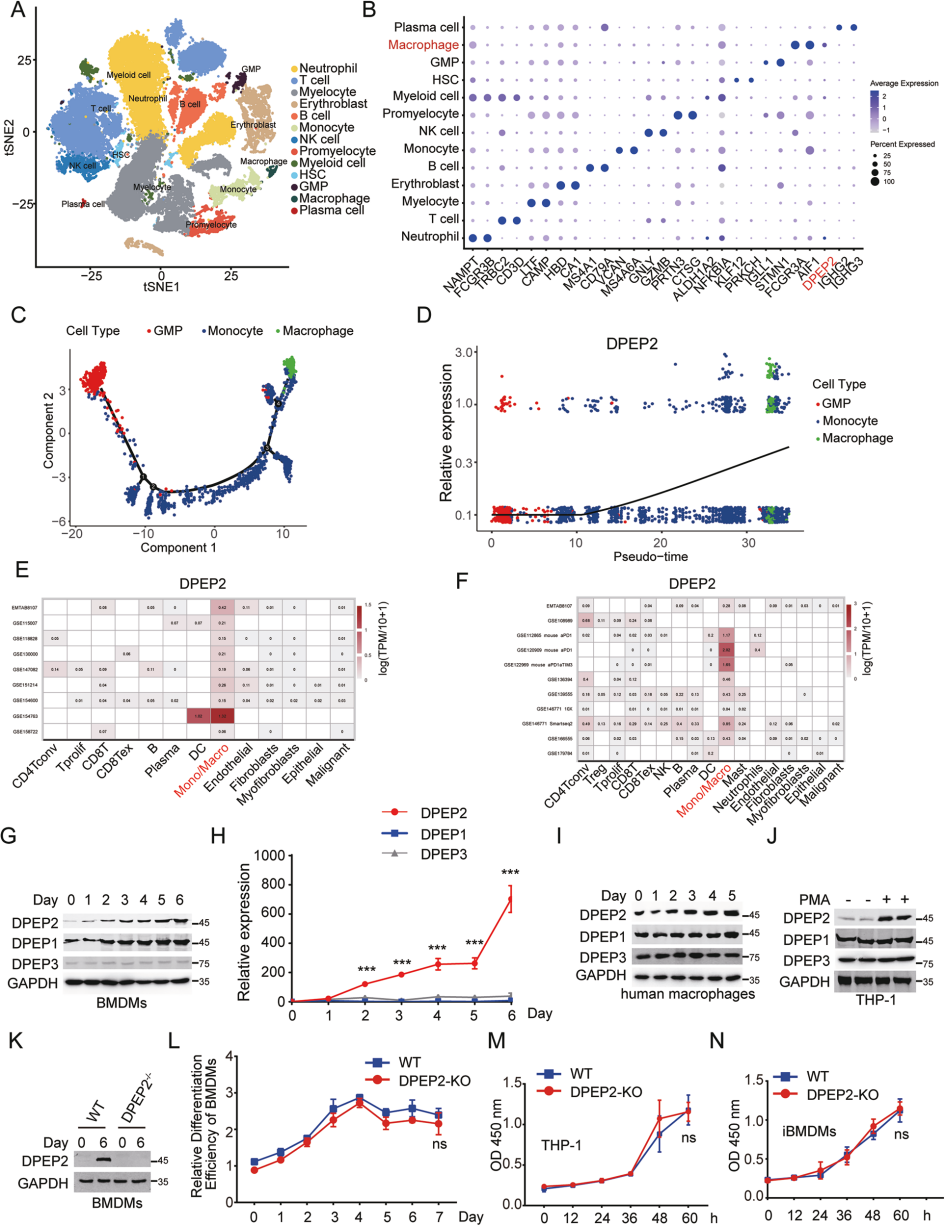
DPEP2 is upregulated in the macrophages. **A** t-SNE diagram shows 13 cell types sorted by single cell transcriptome sequencing in bone marrow tissue provided by two healthy people. **B** Dot Plot showed the expression of the first 2 marker genes of 13 cell types and the gene of interest DPEP2 in each cell type. **C** GMP- Monocyte-macrophage differentiation locus. **D** The expression of DPEP2 increased gradually during the differentiation from GMPs to macrophages. Calculation of DPEP2 expression levels of different cell subpopulations in ovarian tumor tissues (**E**) and colorectal carcinoma tissues (**F**) using the TISCH2 Dataset. The expression of DPEP1/2/3 protein (**G**) and mRNA (**H**) during the differentiation of BMDM from HSCs in vitro. **I** The expression of DPEP1/2/3 protein detected by Western blot assays during the differentiation of human macrophages in vitro. **J** The expression of DPEP1/2/3 protein detected by Western Blot assays during the differentiation of macrophages from THP-1 by PMA treatment in vitro. **K** The expression of DPEP2 protein detected by Western blot assays in BMDMs from WT or DPEP2-KO HSCs in vitro. **L** The relative differentiation efficiency of BMDMs from WT or DPEP2-KO HSCs in vitro. DPEP2 knockout has no influences on the cell vitality of THP-1 (**M**) or iBMDMs (**N**). All data are expressed as mean ± SD. ns denotes no signification. ***P < 0.001.

To evaluate the role of DPEP2 in macrophage-mediated inflammation, we first analysed its expression levels in immune cells in a healthy human bone marrow using a published single-cell sequencing dataset (GSE231946, subsets GSM7306384 and GSM7306386). After cell filtration, 37,997 cells were included in the analysis (Fig. 2A). According to the significant marker genes for each cluster, we integrated the cells into 13 types, including neutrophils, T cells, myelocytes, erythroblasts, B cells, monocytes, NK cells, promyelocytes, myeloid cells, haematopoietic stem cells (HSCs), granulocyte‒monocyte progenitors (GMPs), macrophages, and plasma cells. The expression of DPEP2 differed among the distinct immune cells, and the expression of DPEP2 in macrophages was much greater than that in other immune cells, including monocytes, NK cells, T cells, B cells, plasma cells, and neutrophils (Fig. 2B). Bone marrow-derived macrophages differentiate from monocytes, which are derived from granulocyte‒monocyte progenitors [37]. We subsequently performed a cell trajectory analysis on GMPs, monocytes and macrophages to reconstruct the cell differentiation process (Fig. 2C). DPEP2 was gradually upregulated with the differentiation of macrophages (Fig. 2D), which indicated the potentially important role of DPEP2 in macrophages.

Tissue-resident macrophages also have essential effects on immune-associated diseases [38]. We next analysed DPEP2 expression in distinct immune cells in the tumor microenvironment via an online tool, the TISCH2 dataset (http://tisch.comp-genomics.org). The DPEP2 expression in tumor-resident monocytes/macrophages from colorectal tumor tissues and ovarian serous cystadenocarcinoma tissues was much higher than that in other immune cells (Fig. 2E, F), whereas we did not find increased expression of DPEP1 or DPEP3 in tumor-resident macrophages, which demonstrated the potential function of DPEP2, independent of its dipeptidase activity in macrophages (Fig. S2A–D).

To validate our findings, we determined the expression levels of DPEP2 during the differentiation of mouse BMDMs in vitro. Both the mRNA and protein levels of DPEP2 increased with the differentiation of macrophages, whereas the expression of DPEP1 or DPEP3 protein did not change significantly (Fig. 2G, H). We then differentiated human PBMCs into macrophages using our reported approach [1, 39] and detected alterations in DPEP expression during differentiation. DPEP2, but not DPEP1 or DPEP3, was upregulated during the differentiation of macrophages (Fig. 2I). Moreover, we treated THP-1 cells with PMA to differentiate them into macrophages [40] and found that THP-1-derived macrophages expressed more DPEP2 than nontreated THP-1 cells did (Fig. 2J).

To elucidate whether the DPEP2 gene is essential for the differentiation of macrophages, we generated DPEP2^fl/fl^ Cre^+^ mice by crossing DPEP2^fl/fl^ mice with Cre^+^ mice whose DPEP2 gene was deleted (DPEP2-KO) via tamoxifen treatment. Bone marrow (BM) cells were isolated from WT or DPEP2-KO mice and differentiated into macrophages. There was no difference in the differentiation efficiency between WT and DPEP2-KO BM cells (Fig. 2K, L), which indicated that DPEP2 was not indispensable for the differentiation of macrophages. In addition, DPEP2 deletion had no detrimental effects on immortalized BMDMs (iBMDMs) or THP-1 cells (Fig. 2M, N and S3A, B).

Overall, the data showed that DPEP2 was overexpressed, but not indispensable, in macrophages.

### DPEP2 deficiency enhances the inflammatory response of macrophages

As the expression of DPEP2 was upregulated in macrophages and was inhibited during the activation of macrophages, we assumed that DPEP2 might regulate the proinflammatory effects of macrophages. To verify this hypothesis, macrophage-specific Dpep2-deficient mice (Dpep2^fl/fl^ LyzM-Cre^+^) were generated by crossing Dpep2^fl/fl^ mice with LyzM-Cre^+^ mice (Fig. S3C, D). DPEP2 protein expression was inhibited in monocytes/macrophages from  Dpep2^fl/fl^ LyzM-Cre^+^ mice, whereas the DPEP2 protein was almost undetectable in neutrophils and dendritic cells from WT or Dpep2fl/fl LyzM-Cre+ mice (Fig. S3C, D).

**Fig. 3:**
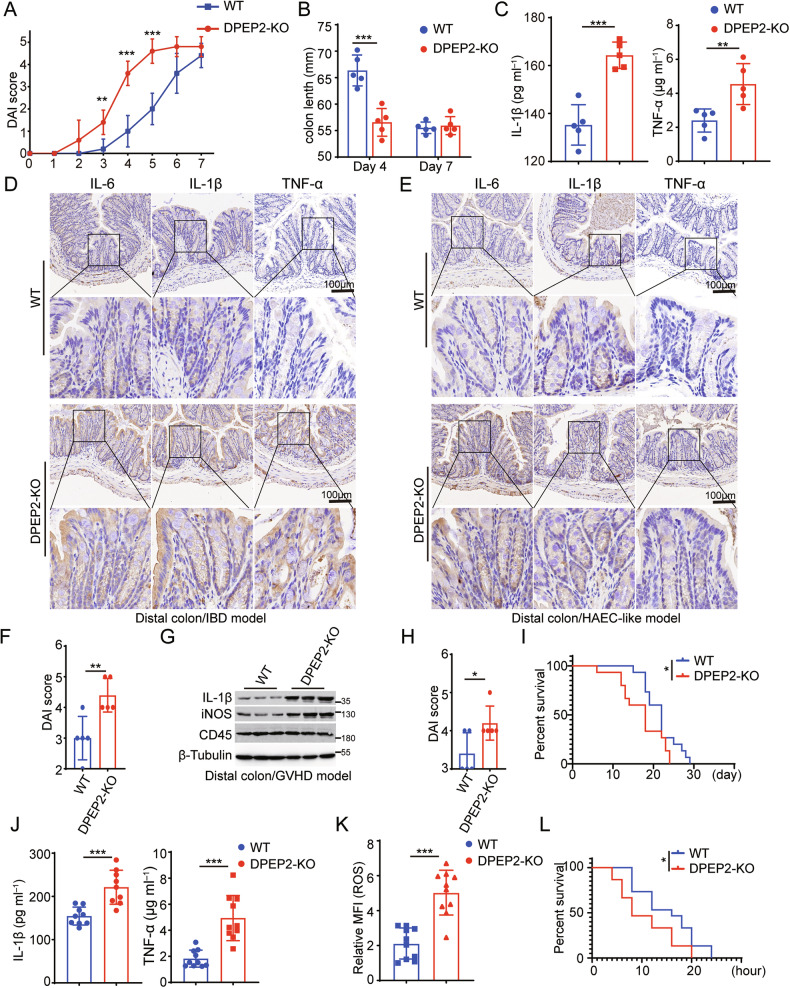
DPEP2 deficiency enhances inflammatory response of macrophages in vivo. The DAI score (**A**) and colon length (**B**) of IBD models using macrophage-specific DPEP2-KO or WT mice at the indicated time points. *n* = 5 per group. The serum (**C**) or colonic (**D**) concentration of inflammatory cytokines in the IBD models using macrophage-specific DPEP2-KO or WT mice. *n* = 5 per group. The colonic concentration of inflammatory cytokines (**E**) and DAI score (**F**) in the HAEC-like models using macrophage-specific DPEP2-KO or WT mice. *n* = 5 per group. The colonic (**G**) concentration of inflammatory cytokines and DAI score (**H**) in the GVHD models using macrophage-specific DPEP2-KO or WT BM cells (C57BL/6) transplanted into irradiated BALB/c mice. *n* = 5 per group. **I** Kaplan-Meier survival curve showed that macrophage-specific Dpep2-deficiency significantly shortened overall survival of the GVHD mice. *n* = 15 per group. **J–L** Macrophage-specific Dpep2-deficiency induced higher serum concentration of inflammatory cytokines, higher ROS production (**J**, **K**, *n* = 10 per group), and shortened overall survival (**K**, *n* = 15 per group) of LPS-induced model of sepsis. All data are expressed as mean ± SD. *P < 0.05, **P < 0.01, and ***P < 0.001.

We further examined the role of DPEP2 deficiency in macrophage-mediated inflammation in multiple models of intestinal inflammation. First, an IBD mouse model was generated by feeding mice with dextran sulfate sodium (DSS) in drinking water (Fig. S3E). A more severe inflammatory response, including higher DAI scores (Fig. 3A), shorter colon lengths (Fig. 3B), and higher serum or colonic concentrations of inflammatory cytokines (Fig. 3C, D), was detected in the macrophage-specific Dpep2-deficient mice than in the wild-type mice. We next established a HAEC-like intestinal inflammation model induced by dysfunctional macrophages, as we reported previously [1] (Fig. S3F) and found that DPEP2 deficiency in macrophages promoted the development of intestinal inflammation. More inflammatory cytokines and higher DAI scores were observed in the macrophage-specific Dpep2-deficient group than in the WT group (Fig. 3E, F). Furthermore, allogeneic graft-versus-host disease (GVHD) impairs intestinal function and induces intestinal inflammation, which is aggravated by M1 macrophage polarization [41]. We transplanted BM cells and splenic T cells from B6-background Dpep2^fl/fl^ LyzM-Cre^+^ or WT mice into irradiated BALB/c mice to establish a GVHD model as described in previous studies [41, 42] (Fig. S3G). Intestinal conditions were analysed on Day 14, and overall survival was recorded. Macrophage-specific Dpep2 deficiency promoted the development of GVHD. Higher expression of inflammatory cytokines and higher DAI scores were observed in the macrophage-specific Dpep2-deficient group than in the WT group (Fig. 3G, H), and macrophage-specific Dpep2 deficiency significantly shortened the overall survival of the GVHD mice (Fig. 3I). Moreover, macrophage-specific Dpep2 deficiency increased the serum concentrations of inflammatory cytokines, higher reactive oxygen species (ROS) production and shortened overall survival in the LPS-induced model of sepsis (Fig. 3J–L).

**Fig. 4:**
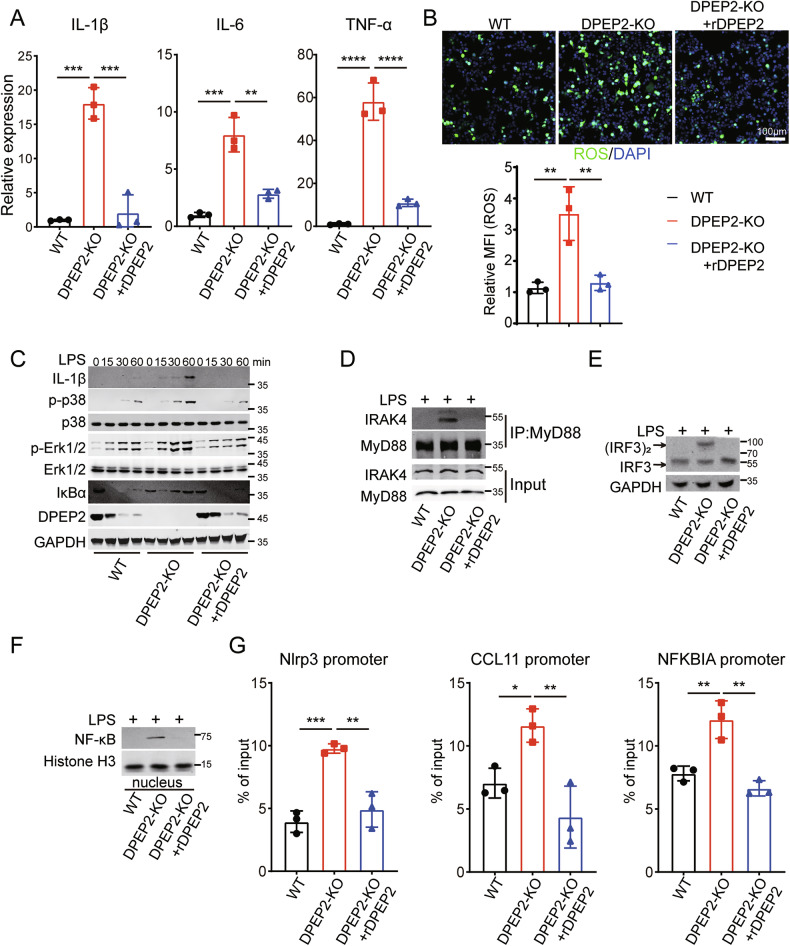
DPEP2 deficiency enhances inflammatory response of macrophages in vitro. **A** mRNA expression levels of inflammatory cytokines (IL-1β, IL-6, and TNF-α) in WT, DPEP2-KO, and DPEP2-KO + DPEP2 re-expression (rDPEP2) iBMDMs treated with LPS treatment (1 μg/ml, 6 h). *n* = 3 per group. **B** ROS production levels in WT, DPEP2-KO, and DPEP2-KO + DPEP2 re-expression (rDPEP2) iBMDMs treated with LPS treatment (1 μg/ml, 6 h). Representative images of ROS production were in up and statistical analysis was in down. *n* = 3 per group. **C** Western blot assays showing IL-1β and MyD88 dependent changes in IκBα levels, p-p38, and p-ERK1/2 at indicated time points of treatment with LPS (1 μg/ml) in WT, DPEP2-KO, and DPEP2-KO+ rDPEP2 iBMDMs. **D** The complex of MyD88 and IRAK4 detected by Co-IP and Western blot analysis 30 min post treatment with LPS (1 μg/ml) in WT, DPEP2-KO, and DPEP2-KO+rDPEP2 iBMDMs. **E** Indicating active (dimerized) IRF3 detected by Western blot assays using NativePAGE electrophoresis 30 min post treatment with LPS (1 μg/ml) in WT, DPEP2-KO, and DPEP2-KO+rDPEP2 iBMDMs. **F** NF-κB expression levels in nucleus detected by nuclear protein extraction and Western blot assays 60 min post treatment with LPS (1 μg/ml) in WT, DPEP2-KO, and DPEP2-KO+ rDPEP2 iBMDMs. **G** The detection of NF-κB activation as an inflammatory transcription factor by ChIP-qPCR targeting Nlrp3, CCL11, and NFKBIA promoter in WT, DPEP2-KO, and DPEP2-KO+ rDPEP2 iBMDMs with LPS treatment. *n* = 3 per group. All data are expressed as mean ± SD. *P < 0.05, **P < 0.01, and ***P < 0.001.

We further investigated the role of DPEP2 in the activation of macrophages using multiple in vitro models. DPEP2 deletion enhances the expression of inflammatory cytokines and the production of ROS in macrophages responding to inflammatory stimulation, that can be rescued by re-expression of DPEP2 (Fig. 4A–C). In addition, DPEP2 deletion promoted the transduction of multiple key inflammatory signals, including enhanced activation of the TRIF-mediated and TIRAP/MyD88-mediated signaling pathways (Fig. 4C–E). Increased translocation of NF-κB into the nucleus (Fig. 4F) and increased transcription of the inflammatory factor NF-κB (Fig. 4G) were also observed in DPEP2-KO macrophages. All the effects of DPEP2 deletion were abrogated by the re-expression of DPEP2 (Fig. 4C–G).

Taken together, DPEP2 deficiency promotes the development of intestinal inflammation by enhancing the activation of macrophages. More investigations are needed to determine the mechanisms by which DPEP2 deletion promotes macrophage-mediated inflammation.

### DPEP2 downregulation triggers the NF-κB and p38 pathways by releasing MAP3K7 in activated macrophages

**Fig. 5:**
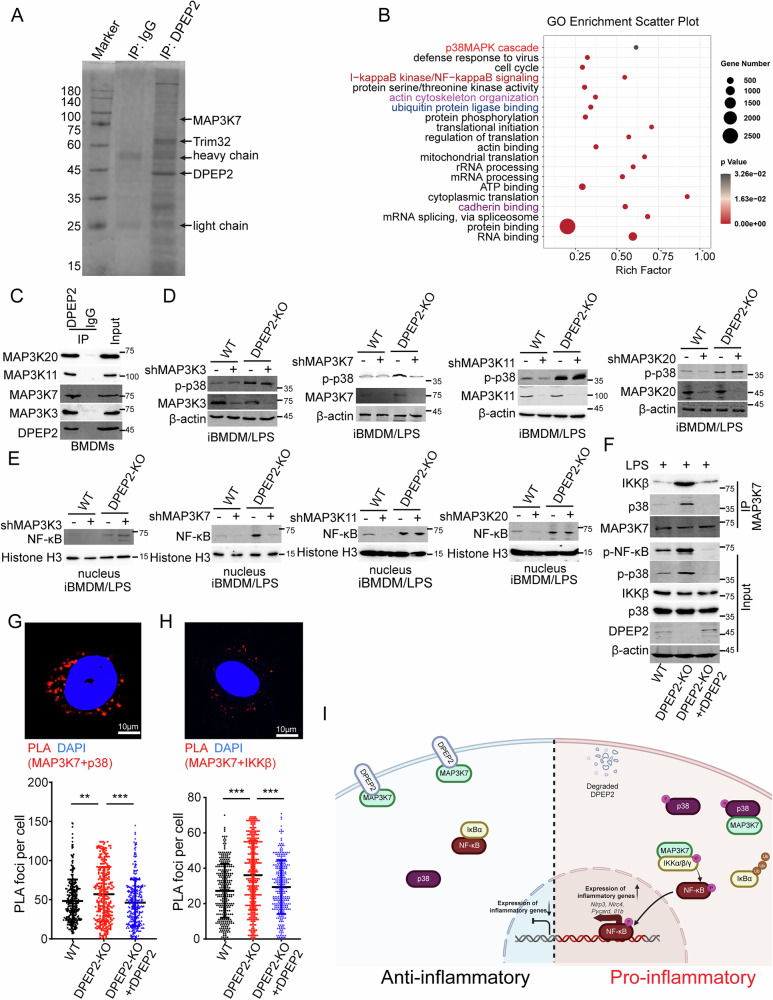
DPEP2 downregulation triggers NF-κB and p38 pathways by releasing MAP3Ks in activated macrophages. **A** DPEP2 and DPEP2-associated proteins from BMDM cells were isolated using DPEP2 antibodies. IgG antibodies were set as negative control. The eluates were resolved by SDS/PAGE, subjected to silver staining, and analyzed by mass spectrometry. Key proteins are labeled by the protein names. **B** GO functional enrichment analysis of DPEP2-associated proteins detected by mass spectrometry. The DPEP2-associated proteins were also listed in Table S5. **C** Co-IP assays identified the interaction of DPEP2 with MAP3K3, MAP3K7, MAP3K11, and MAP3K20 in BMDMs. Repression of MAP3K7 but not MAP3K3, MAP3K11, or MAP3K20 inhibits DPEP2 KO-mediated activation of p38 MAPK (**D**) and NF-κB (**E**) signals in BMDMs. **F** DPEP2 deletion increases the interaction of MAP3K7 with IKKβ and p38 and enhances phosphorylation of NF-κB and p38, and rDPEP2 impairs the interaction of MAP3K7 and IKKβ or p38 proteins. The interaction of MAP3K7 with p38 (**G**) and IKKβ (**H**) in WT, DPEP2-KO, and DPEP2-KO+rDPEP2 iBMDMs were detected using PLA assays. Representative images of PLA foci were in up and statistical analysis was in down. *n* = 300 per group. **I** Pattern diagram of the inflammatory role of DPEP2 in macrophages. overexpression of DPEP2 silences inflammatory signal transduction by inhibiting MAP3K7 in inactivated macrophages, and DPEP2 downregulation promotes the transduction of NF-κB and p38 MAPK signal by releasing MAP3K7 in pro-inflammatory macrophages. The image was created by biorender.com. All data are expressed as mean ± SD. **P < 0.01, and ***P < 0.001.

Among immune cells, DPEP2 was specifically expressed in macrophages, but Dpep2 deficiency enhanced the proinflammatory function of macrophages. Moreover, the DPEP2 protein was downregulated during the activation of macrophages (Fig. 1); therefore, we suspected that the downregulation of DPEP2 might lead to a switch of a proinflammatory signal in activated macrophages. To determine the mechanism by which DPEP2 downregulation promotes inflammatory signal transduction in macrophages, MS analysis was performed following Co-IP assays of the DPEP2 protein to identify DPEP2-binding partners (Fig. 5A). As shown in Fig. 5A and Table S5, MS confirmed multiple proteins that DPEP2 binds (Fig. 5B), GO analysis revealed that DPEP2 interacts with various proteins related to acttin cytoskeleton organization and cadherin binding (Fig. 5B), indicating the essential effects of DPEP2 on the cell infiltration, an important feature of proinflammatory macrophages. Further analysis of the MS data identified that DPEP2 also bound multiple proteins that were related to the NF-κB and p38 pathways (Fig. 5B). Moreover, it was found that DPEP2 interacted with 4 members of the mitogen-activated protein kinase kinase kinase (MAP3K) family, MAP3K3, MAP3K7, MAP3K11, and MAP3K20, which was confirmed using Co-IP assay, followed by western blotting (Fig. 5C and S4A).

MAP3Ks participate in various biological processes in cells, including inflammatory signal transduction, cell proliferation, and the DNA damage response [43–48]. All the MAP3Ks (MAP3K3, MAP3K7, MAP3K11, and MAP3K20) that interacted with DPEP2 act as key activators of NF-κB or p38 MAPK signalings during the inflammatory response [49–53]. To confirm the mechanism by which DPEP2 deficiency enhances the transduction of NF-κB and p38 MAPK signalings (Fig. 4), we knocked down the expression of the MAP3Ks (MAP3K3, MAP3K7, MAP3K11, and MAP3K20) and tested the effects of their knockdown on DPEP2-KO-mediated activation of NF-κB and p38 MAPK signalings. The MAP3K7 KD neutralized the promoting effects of DPEP2 deletion on NF-κB and p38 MAPK signalings (Fig. 5D, E). MAP3K7, also termed TAK1, has been identified as an essential activator of the NF-κB and p38 MAPK pathways [54–56]. In addition, DPEP2 deletion increased the interaction of MAP3K7 with IKKβ and p38 and increased the phosphorylation of NF-κB and p38, while DPEP2 re-expression impaired the interaction of MAP3K7 with the IKKβ and p38 proteins (Fig. 5F). PLA also revealed that DPEP2 suppression increased the interaction of MAP3K7 with the IKKβ and p38 proteins and that DPEP2 re-expression impaired the interaction of MAP3K7 with the IKKβ and p38 proteins (Fig. 5G, H). These data demonstrated that DPEP2 impaired MAP3K7-mediated activation of NF-κB and p38 MAPK signaling and that DPEP2 downregulation promoted inflammatory signal transduction.

Overall, overexpression of DPEP2 silences inflammatory signal transduction by inhibiting MAP3K7 in inactivated macrophages, and DPEP2 downregulation promotes the transduction of NF-κB and p38 MAPK signaling by releasing MAP3K7 in proinflammatory macrophages (Fig. 5I).

### DPEP2 is degraded by Trim32-mediated ubiquitination in activated macrophages

**Fig. 6:**
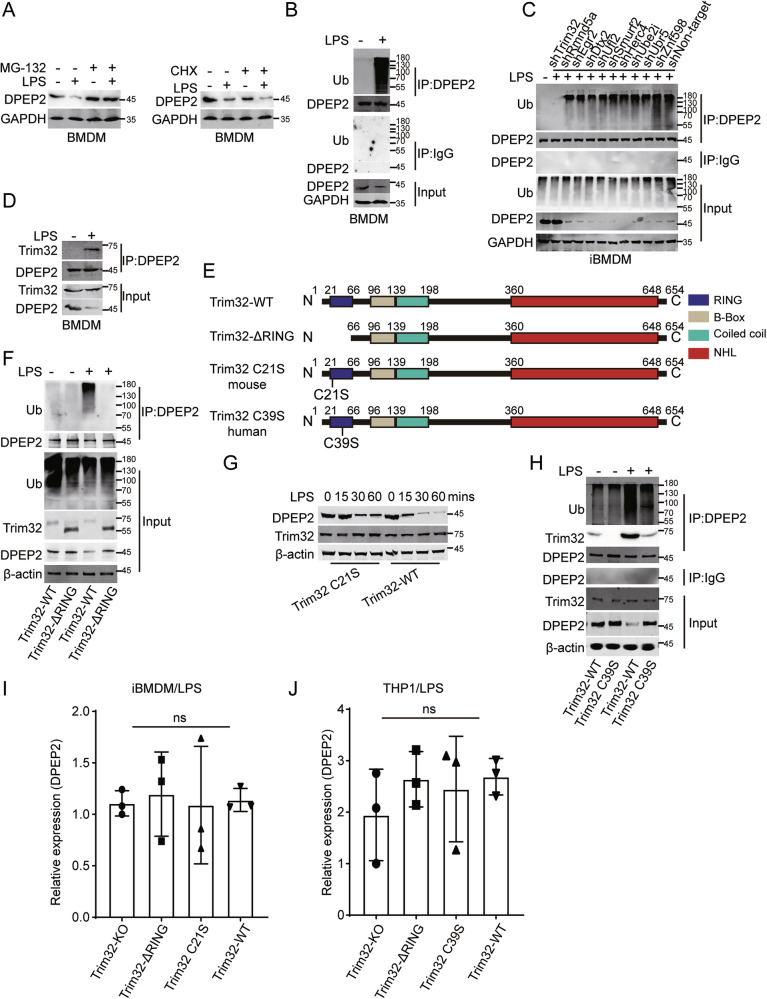
DPEP2 is degraded by Trim32-mediated ubiquitination modification in activated macrophages. **A** MG132 treatment but not CHX inhibited the downregulation of DPEP2 in LPS-treated macrophages. **B** LPS treatment induces increased DPEP2 ubiquitination in macrophages. **C** Knockdown of Trim32 but not Rmnd5a, Egr2, Dtx2, Ufl2, Smurf2, Herc4, Ube2i, Ubr5 or Znf598 repress the DPEP2 ubiquitination in activated macrophages. **D** More interaction of DPEP2 and Trim32 protein was detected in the activated pro-inflammatory macrophages. **E** Pattern diagram of Trim32-WT, Trim32-ΔRING, Trim32 C21S and Trim32 C39S protein. **F** Trim32-ΔRING decreases the ubiquitination of DPEP2 and stabilizes DPEP2 proteins responding to inflammatory stimulation in macrophages. RING-deficient Trim32, Trim32 C21S (**G**) or Trim32 C39S (**H**), downregulated both ubiquitination and degradation of DPEP2. Repression the catalytic activity of Trim32 as an E3 ubiquitin-protein ligase has no influence on the mRNA levels of DPEP2 in iBMDMs (**I**) and THP-1 (**J**). All data are expressed as mean ± SD. ns denotes no signification.

Since DPEP2 downregulation is essential for activating macrophages, more investigations are necessary to explore the molecular processes that regulate the DPEP2 protein. MG132 (a proteasomal inhibitor) and cycloheximide (CHX; an inhibitor of protein synthesis) were used to study the regulation of the DPEP2 protein. MG132 treatment inhibited the downregulation of DPEP2 in LPS-treated macrophages; however, CHX had no significant effect on DPEP2 regulation in LPS-treated macrophages (Fig. 6A), indicating that DPEP2 downregulation may result from increased DPEP2 degradation in proinflammatory macrophages. In addition, assays were performed to detect posttranslational modification (PTM) of DPEP2 (Fig. 6B and S4B). Ubiquitination of DPEP2 was increased in macrophages in response to inflammatory stimulation (Fig. 6B). Next, we confirmed the causation of DPEP2 ubiquitination in response to inflammatory stimulation by further analysing MS data, and several ubiquitination-related molecules, including Trim32, Rmnd5a, Egr2, Dtx2, Ufl2, Smurf2, Herc4, Ube2i, Ubr5 and Znf598 (Table S5), were shown to bind DPEP2. To investigate the molecules that target DPEP2 in proinflammatory macrophages, we knocked down the indicated proteins and tested the levels of DPEP2 ubiquitination (Fig. S4C). The suppression of the E3 ubiquitin-protein ligase tripartite motif-containing protein 32 (Trim32) impaired DPEP2 ubiquitination and increased the protein expression of DPEP2 in activated macrophages (Fig. 6C), indicating that DPEP2 may be degraded by Trim32-mediated ubiquitination.

Direct interactions between DPEP2 and Trim32 were detected in macrophages and were validated via the ectopic expression of DPEP2 and Trim32 in HEK293T cells (Figs. 6D and S4D). More interactions between DPEP2 and Trim32 were detected in activated proinflammatory macrophages (Fig. 6D). In addition, the RING domain, which is essential for the catalytic activity of E3 ubiquitin-protein ligases [24, 57], was removed from Trim32 to determine the role of Trim32 in DPEP2 ubiquitination (Fig. 6E). Deleting the RING domain of Trim32 (Trim32-ΔRING) decreased the ubiquitination of DPEP2 and stabilized the DPEP2 protein in response to inflammatory stimulation of macrophages (Fig. 6F). We further mutated the Cys21 residue to a Ser residue in the RING motif of mouse Trim32 and the Cys39 residue to a Ser residue in the RING motif of human Trim32 [28, 29], which impaired their secondary structures and generated RING-deficient Trim32 proteins (Trim32 C21S and Trim32 C39S, respectively) (Fig. 6E). Both the ubiquitination and degradation of DPEP2 in response to inflammatory stimulation were lower in macrophages with the RING-deficient Trim32 proteins than in those with WT Trim32 proteins (Fig. 6G, H). Moreover, the suppression of Trim32 had no influence on the mRNA levels of DPEP2 in macrophages in response to inflammatory stimulation (Fig. 6I, J).

**Fig. 7:**
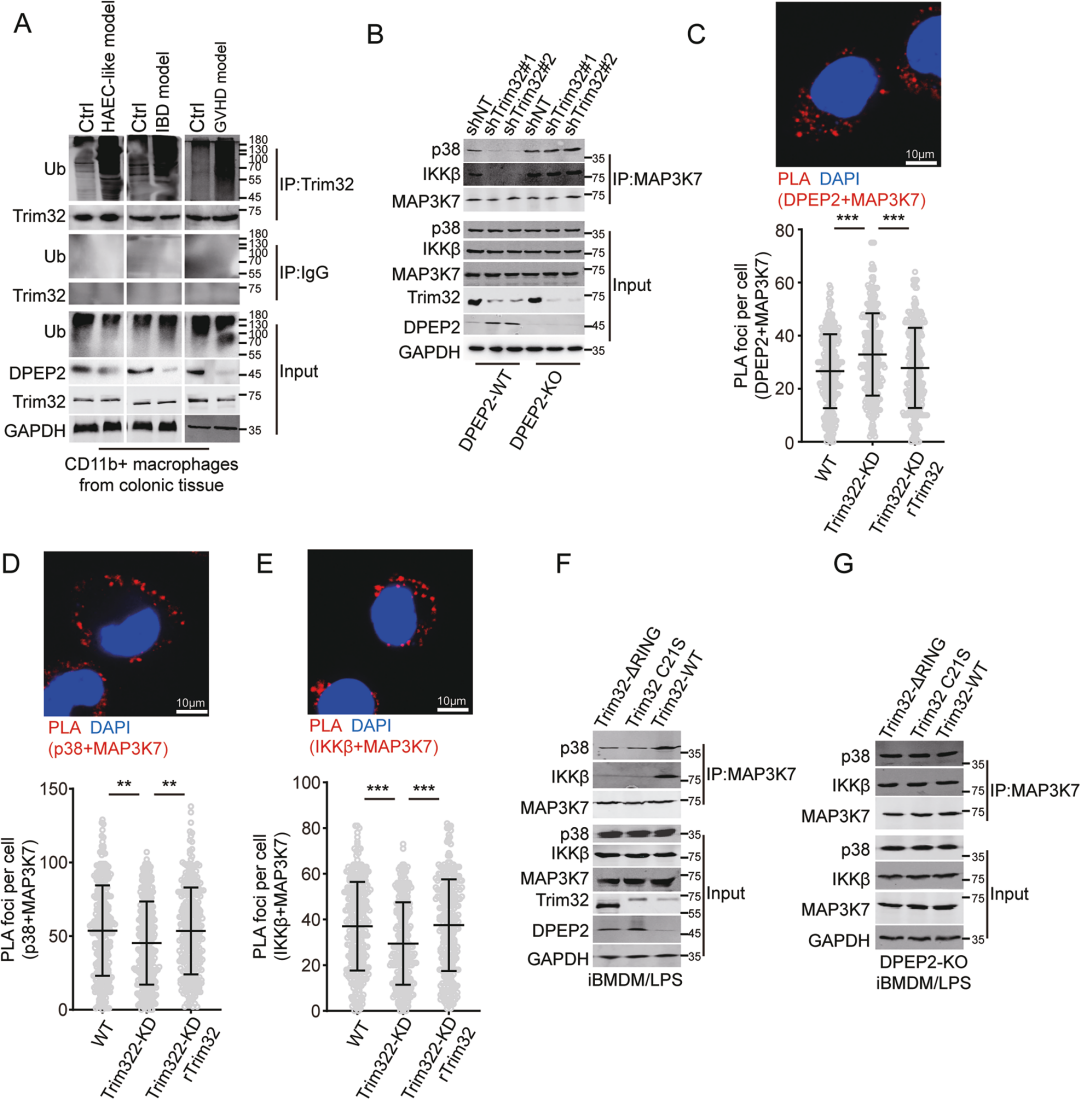
Inhibition of Trim32 represses transduction of NF-κB and p38 signalings by DPEP2-mediated MAP3K7 inactivation. **A** Ubiquitination levels of Trim32 were increased in colonic macrophages from mouse model of IBD, GVHD, and HAEC like intestinal inflammation. **B–E** Trim32 increases the interaction of MAP3K7 with DPEP2 and decreases interaction of MAP3K7 with IKKβ and p38 by increasing the expression of DPEP2 in macrophages with LPS. Co-IP assays detecting the interaction of MAP3K7 and p38 or IKKβ were in (**B**). PLA assays of interaction of MAP3K7 and DPEP2 were in (**C**). *n* = 300 per group. PLA assays of interaction of MAP3K7 and p38 were in (**D**). *n* = 300 per group. PLA assays of interaction of MAP3K7 and IKKβ were in (**D**). *n* = 300 per group. The effects of RING-deficiency of Trim32 on the interaction of MAP3K7 with IKKβ and p38 in the WT (**F**) or DPEP2-KO (**G**) iBMDMs. All data are expressed as mean ± SD. **P < 0.01, and ***P < 0.001.

Overall, these results suggest that DPEP2 is ubiquitinated and stabilized by Trim32 in macrophages in response to inflammatory stimulation.

### Inhibition of Trim32 suppresses inflammatory signal transduction by DPEP2-mediated MAP3K7 inactivation

Self-ubiquitination is essential for the activation of Trim32, an E3 ubiquitin-protein ligase [29]. Ubiquitination of Trim32 was increased in colonic macrophages from multiple models of intestinal inflammation, including the IBD, GVHD, and HAEC-like intestinal inflammation models (Fig. 7A). Although Trim32 suppression increased the expression of DPEP2, it has no significant influence on the activation of NF-κB or p38 signaling or inflammatory responses to LPS treatment in macrophages via multiple Trim32-mediated inflammatory pathways (Fig. S4E, F). Trim32 suppression increased the interaction of MAP3K7 with DPEP2 and decreased the interaction of MAP3K7 with IKKβ and p38 by stabilizing DPEP2 in activated macrophages, but these effects were neutralized by Trim32 reexpression (Fig. 7B–E). In addition, both Trim32-ΔRING and Trim32 C21S but not Trim32 WT impaired the interaction of MAP3K7 with IKKβ and p38 by stabilizing DPEP2, and these effects were blocked by DPEP2 deletion in macrophages (Fig. 7F, G). These data demonstrate that DPEP2 acts as a key bridge for Trim32-mediated activation of NF-κB and p38 MAPK signalings.

**Fig. 8:**
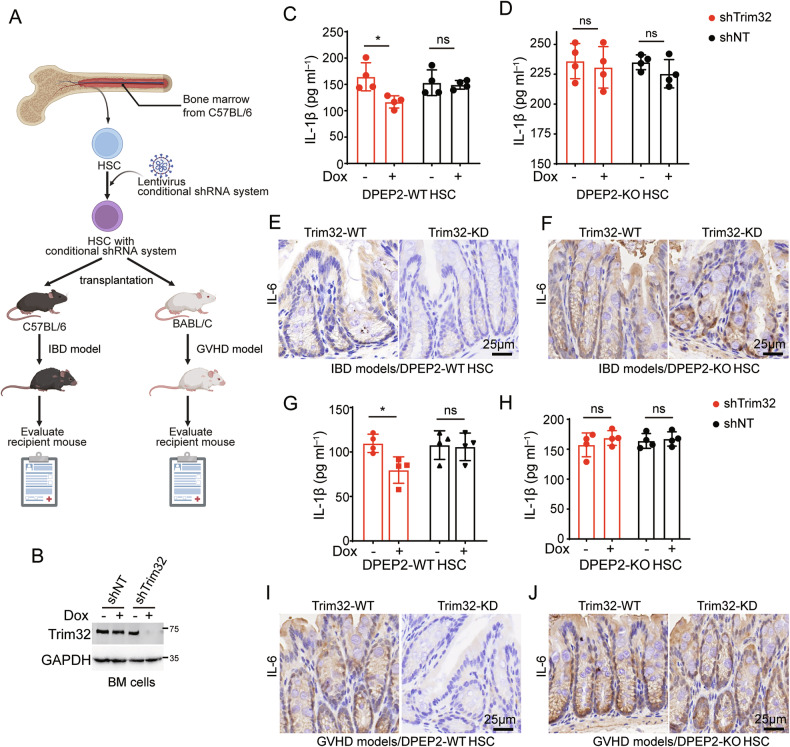
Inhibition of Trim32 represses development of inflammation by DPEP2 in vivo. **A** Pattern diagram of IBD and GVHD models with Trim32 KD BM cells. B6-background BM cells transfected with a conditional shRNA system targeting Trim32 were transplanted into irradiated myeloablative mice. IBD and GVHD models were established using recipient mice to detect the role of Trim32. The image was created by biorender.com. **B** Trim32 knockdown using a conditional shRNA system in BM cells with or without Dox treatment. Trim32 KD in HSCs decreases the expression of serum and colonic proinflammatory cytokines in IBD models (**C**, **E**, *n* = 4 per group), which is blocked by DPEP2 deletion (**D**, **F**, *n* = 4 per group). Trim32 KD in HSCs decreases the expression of serum and colonic proinflammatory cytokines in GVHD models (**G**, **I**, *n* = 4 per group), which is blocked by DPEP2 deletion (**H**, **J**, *n* = 4 per group). All data are expressed as mean ± SD. *P < 0.05, ns denotes no signification.

To validate our findings, B6-background BM cells that were transfected with a conditional shRNA system targeting Trim32, in which Trim32 was knocked down with doxycycline (Dox) treatment, were transplanted into irradiated myeloablative mice (Fig. 8A, B). BM cells with a nontargeting shRNA system were used as the negative control group. an IBD mouse model was generated by feeding mice with dextran sulfate sodium (DSS) in drinking water (Fig. 8A). Trim32 suppression via pretreatment with Dox decreased the expression of serum or colonic proinflammatory cytokines in the IBD model, but this effect was blocked by DPEP2 deletion (Fig. 8C–F). Next, B6-background BM cells transfected with the conditional shRNA system targeting Trim32 were transplanted into irradiated BALB/c mice to generate a GVHD model (Fig. 8A). Similar to the above results, Trim32 inhibition by pretreatment with Dox decreased the expression of proinflammatory cytokines in the GVHD model, but this effect was reversed by DPEP2 deletion (Fig. 8G–J), indicating that Trim32 enhances the activation of macrophages by destabilizing DPEP2.

Taken together, these findings indicate that Trim32 destabilizes DPEP2 and then enhances VIM activation and MAP3K7-mediated activation of NF-κB and p38 MAPK signalings, which results in increased infiltration and rapid activation of proinflammatory macrophages. Targeting the Trim32-DPEP2 axis may lead to innovative therapeutic strategies for various types of macrophage-mediated intestinal inflammation.

## Discussion

The prevalence of intestinal inflammation has increased with the progress of society. The development of intestinal inflammation is influenced by multiple factors, including genetic [58], environmental [59, 60], microbiota-related [61, 62], immunological [4, 63], and mental [64] factors. Additionally, it has been reported that macrophages are involved in the process of intestinal inflammation [4, 5, 8], which makes them a potential therapeutic target.

In this study, the proteomic and transcriptomic data of LPS-V-treated macrophages were analysed using MS analysis and RNA sequencing. The discrepancies between the proteomic and transcriptomic data were further investigated. DPEP2 was found to be overexpressed in macrophages but not in other immune cells; however, DPEP2 knockout had no detrimental effects on macrophages. In addition, the protein level of DPEP2 was downregulated during the activation of macrophages. However, inflammatory stimulation had no influence on the mRNA level of DPEP2. Additional investigations revealed that DPEP2 deletion enhanced macrophage-mediated inflammation in vivo and in vitro, which could be reversed by DPEP2 re-expression. These findings indicate an essential role of DPEP2 in the inflammatory activation of macrophages.

The DPEP family members DPEP1, DPEP2, and DPEP3 are dipeptidases that hydrolyse a wide range of dipeptides [22], including the conversion of leukotriene D4 to leukotriene E4. In addition, several proinflammatory effects of DPEPs, independent of their dipeptidase activity, have been revealed in previous studies [18, 19, 21]. Aberrant expression of DPEP2 has been observed in various inflammation-related diseases [20, 21, 23], but the molecular function of DPEP2 in inflammation remains obscure.

Dpep2^fl/fl^ LyzM-Cre^+^ mice were introduced into this study as macrophage-specific Dpep2-deficient mice to investigate the effects of Dpep2 on the development of intestinal inflammation. Macrophage-specific Dpep2 deficiency promoted the development of intestinal inflammation. The conditional Lys-M-Cre mouse model is one of the most common models used for investigating macrophages in vivo [65, 66]. However, the Lys-M gene is expressed in macrophages, neutrophils, and dendritic cells, which results in limitations in macrophage-specific Dpep2-deficient mice. In this study, the DPEP2 protein was undetectable in WT neutrophils and dendritic cells, indicating the minimal influence of Dpep2 deficiency on neutrophils and dendritic cells. Therefore, Dpep2^fl/fl^ LyzM-Cre^+^ mice could be used as macrophage-specific Dpep2-deficient mice to support the conclusions of this study.

DNA is a script, and proteins are the actors of biology. Following the central dogma, proteins are translated from mRNA, which is transcribed on the basis of DNA. Most genes are regulated by controlling the level of mRNA. The alteration of mRNA subsequently results in parallel regulation of proteins. However, some biological processes, such as the modifications of mRNAs and proteins, break the abovementioned rules concerning gene regulation [67, 68]. Moreover, the discrepancy between proteomic and transcriptomic data is essential but often neglected. For example, the Hif-1a protein is stabilized via a reduction of the hydroxylation modification in response to hypoxia [69], and the Nrf2 protein accumulates and translocates into the nucleus after its release from Keap1-mediated degradation in response to inflammatory stimulation or itaconic acid treatment [17, 70]; neither of these processes influences the corresponding mRNAs. Direct regulation of proteins can be achieved through independent transcription and translation. In this study, we found that DPEP2 was destabilized via Trim32-mediated degradation immediately following the activation of Trim32 (autoubiquitination) in response to inflammatory stimulation. Additionally, DPEP2 degradation released MAK3K7 and subsequently enhanced the activation of p38 MAPK and NF-κB signalings. Taken together, DPEP2 is overexpressed and arrests abundant MAK3K7 in inactivated macrophages, and the release of MAK3K7 by the degradation of DPEP2 promotes the activation of p38 MAPK and NF-κB signalings in response to stimulation, resulting in the acute transduction of inflammatory signals in macrophages.

The transduction of inflammatory signals in macrophages is very complex. In this study, differentially expressed proteins and mRNAs were identified using MS analysis and RNA sequencing. Owing to the limitations of MS analysis, the number of differentially expressed proteins detected by MS analysis was much lower than that of differentially expressed mRNAs detected using RNA sequencing. The lower sensitivity of MS analysis resulted in false-negative results for various proteins that were reported to be regulated in activated macrophages in previous studies [17, 71, 72]. Modified MS analysis with increased sensitivity is necessary for detecting the direct regulation of proteins.

In conclusion, overexpressed DPEP2 inhibits the transduction of inflammatory signals by assisting MAK3K7 in inactivated macrophages. Moreover, DPEP2 is ubiquitinated and degraded by activated Trim32, which results in strong transduction of inflammatory signals through the release of MAK3K7 in proinflammatory macrophages. Thus, the Trim32-DPEP2 axis may be a potential therapeutic target for the treatment of intestinal inflammation and for the modification of macrophage-mediated cytotherapy.

## Methods

### Cell lines

The murine RAW 264.7, THP-1 and HEK293T cell lines were obtained from Stem Cell Bank, Chinese Academy of Sciences. Immortalized Bone Marrow-Derived Macrophages (iBMDM) were purchased from Mcellbank (Shanghai). All cells were cultured in indicated medium at 37 °C with 5% CO_2_. All cell lines were tested to be mycoplasma-free and authenticated using short tandem repeat (STR) DNA fingerprinting at Shanghai Biowing Applied Biotechnology Co., Ltd. (Shanghai, China).

### Plasmids

The coding sequences of Dpep1, Dpep2, Dpep3 were amplified from the total DNA of RAW 264.7 or HEK293T cells, sequenced, and subcloned into pLenti-Blast or Lenti-puro plasmids. The shRNA plasmids and lenti-puro plasmids expressing Trim32, MAP3K3, MAP3K7, MAP3K11 and MAP3K20 were purchased from DNA core in Shanghai Jiao Tong University or Saiheng Biotech (Shanghai). Point mutations were generated using a Site-Directed Mutagenesis Kit (Invitrogen) following the manufacturer’s protocol.

### Antibodies and other reagents

Anti-DPEP1 (84292, 87223), anti-P-NF-κB(Ser536) (3033), anti-NF-κB (8242), anti-IRF-3 (11904), anti-P-Erk1/2 (Thr202/Tyr204) (9101), anti-Erk1/2 (9102), anti-P-p38 (Thr180/Tyr182) (4511), anti-p38 (9212), anti-IRAK4 (4363), anti-Cre recombinase (15036), anti-phos-S/T and anti-β-actin (5125) antibodies were purchased from Cell Signaling Technology. Anti-PAR (MABE1031), anti-MAR (MABE1076), anti-Ac-lys (SAB5600275) antibodies were purchased from Millipore. Anti-DPEP3 (ab235451), anti-MyD88 (ab 133739), anti-IκBα (ab32518), anti-CD45 (ab317446) antibodies were obtained from Abcam. Anti-MAP3K3(TP72913), anti-MAP3K7 (T57057), anti-MAP3K11 (PS09111), anti-MAP3K20 (TS11491), anti-phos-S/T (T91067), anti-Trim32 (PS12341), anti-flag (M20008), anti-HA (M20003), anti-V5 (T40006) and anti-GAPDH (M20006) antibodies were obtained from Ab-mart. Anti-Ub (sc-53509) antibodies were purchased from Santa Cruz biotechnology. Anti-CD11b (12-0112) and anti-DPEP2 (PA5-100487) antibodies were purchased from Invitrogen. Anti-DPEP2 (16466-1-AP), Anti-Histone H3 (17168-1-AP) and anti-β-Tubulin (80713-1-RR) antibodies were purchased from Proteintech. Anti-IL6 (GB11117), anti-TNF-α (GB11188) and anti-iNOS (GB11119) antibodies were purchased from Servicebio. Anti-IL1β (AF-401-NA) antibodies were purchased from R&D. Anti-mouse (7076) and anti-rabbit (7074) secondary antibodies were from Cell Signaling Technology. Reagents were purchased from the following sources: Macrophage colony stimulating factor (M-CSF) (300-25) was from peprotech. DSS (60316ES) were from YEASEN. Dox (ST039) and LPS (*E. coli O111:B4*) (S1732) were from Beyotime Biotechnology. CHX (HY-12320) were obtained from Med Chem Express. MG132 (S2619) were obtained from Selleck.

### Mice

The wild type, Dpep2^fl/fl^, LyzM-Cre^+^ and Cre^+^ C57BL/6 mice were purchased from the Gempharmatech Co., Ltd (Nanjing). The macrophage-specific Dpep2-deficient (Dpep2^fl/fl^LyzM-Cre^+^) mice were obtained by crossing Dpep2^fl/fl^ and LyzM-Cre^+^ mice, and the Dpep2-deficient mice were generated by crossing Dpep2^fl/fl^ and Cre+ mice. The WT BABL/c mice were purchased from the Gempharmatech Co., Ltd. The animal protocol was approved by the Ethical Committee of Animal Experiments of Shanghai Children’s Medical Center (SCMC-LAWEC-2021-063). The sample size was estimated according to previous studies and 3 R principle [1, 41, 59]. The mice were allocated randomly into different groups. All animal experiments were performed in accordance with the relevant laws and guidelines of the Institutional Animal Care and Use Committee (IACUC) of Shanghai Jiao Tong University.

### Generation of BMDMs

Generation of BMDMs was performed using our approach described previously [1]. Briefly, bone marrow (BM) cells were isolated from femurs of female C57BL/6 mice (8 weeks), treated with ACK lysis buffer for 5 min to remove red blood cells, centrifuged for 5 min at 300 × *g*, and resuspended in BMDM medium (DMEM supplemented with 10% v/v heat-inactivated FBS, 2 mM L-glutamine, 100 units/mL, 100 μg/mL penicillin/streptomycin, 0.5 mM sodium pyruvate and 100 ng/ml M-CSF). Cells were differentiated for 7 days and then replated for subsequent experiments.

### Isolation of human peripheral blood mononuclear cells (PBMCs)

Human PBMCs were isolated from human blood using Ficoll (GE) as described previously [1]. The whole blood was obtained from Shanghai Children’s Medical Center with written informed consent approved by Medical Research Ethics Committee of Shanghai Children’s Medical Center. Whole blood was mixed with RPMI1640 (1:1), layered on 10 ml Ficoll and spun for 20 min at 2000 rpm. The PBMCs were isolated from the middle layer. PBMCs were washed and then maintained in RPMI 1640 supplemented with 10% FBS, 2 mM L-glutamine, and 1% penicillin/streptomycin solution.

### Generation of human macrophages

CD14 positive cells were isolated from PBMCs magnetic-activated cell sorting (MACS) CD14 beads and then plated at 0.5 × 10^6^ cells/ml with RPMI 1640 supplemented with 10% FBS, 2 mM L-glutamine, 100 μg/mL penicillin/streptomycin and 100 ng/ml human M-CSF. After 5 days of differentiation, human macrophages were counted and replated at 0.5 × 10^6^ cells/ml for subsequent experiments.

### RNA sequencing

Total RNA was extracted by RNeasy Micro Kit (Qiagen, 74004) and then quantified. RNA library construction and sequencing were performed by OE Biotech (Shanghai) Co., Ltd, following the manufacturer’s instructions (Illumina). Briefly, ribosomal RNA was removed using TruSeq Stranded Total RNA with Ribo-Zero for Humans (Illumina). First-strand cDNA was synthesized by random hexamer-primed Superscript II Reverse Transcriptase (Invitrogen), followed by second-strand cDNA synthesis using RNase H and DNA polymerase and ligation of sequencing adapters using a TruSeq RNA LT Sample Prep Kit v2 (Illumina). Then, 50-bp single-end sequencing was conducted using. The raw data are available in SRA database (PRJNA1182745).

### Mass spectrometry (MS)-based quantitative (4D-DIA) proteomics analysis

The peptides were collected after protein extraction from cell powder and trypsin digestion. Then, the tryptic peptides were dissolved in solvent A, directly loaded onto a home-made reversed-phase analytical column (25-cm length, 100 μm i.d.). The mobile phase consisted of solvent A (0.1% formic acid, 2% acetonitrile/in water) and solvent B (0.1% formic acid, 90% acetonitrile/in water). Peptides were separated with the following gradient: 0–22.5 min, 6–22%B；22.5–26.5 min, 22–34%B；26.5–28.5 min, 34–80%B；28.5–30 min, 80%B, and all at a constant flow rate of 700 nl/min on an EASY-nLC 1200 UPLC system (ThermoFisher Scientific). The separated peptides were analyzed in Orbitrap Exploris 480 with a nano-electrospray ion source. The electrospray voltage applied was 2300 V. FAIMS compensate voltage (CV) was set as-45 V. Precursors and fragments were analyzed at the Orbitrap detector. The full MS scan resolution was set to 60,000 for a scan range of 350–1400 m/z. The MS/MS scan was fixed first mass as 120.0 m/z at a resolution of 15000. The HCD fragmentation was performed at a normalized collision energy (NCE) of 27%. Automatic gain control (AGC) target was set at 1E6, with a maximum injection time of 22 ms. “ The DIA data were processed using DIA-NN search engine (v.1.8). Tandem mass spectra were searched against Mus_musculus_10090_SP_20230103.fasta (17132 entries) concatenated with reverse decoy database. Trypsin/P was specified as cleavage enzyme allowing up to 1 missing cleavages. Excision on N-term Met and carbamidomethyl on Cys were specified as fixed modification. FDR was adjusted to < 1%. The mass spectrometry proteomics data have been deposited to the ProteomeXchange Consortium via the PRIDE [73] partner repository with the dataset identifier PXD057405.

### Western blotting and immunoprecipitation (IP) assays

Western blotting and IP assays were performed as our previous description [67]. Briefly, tissues or cells were lysed in RIPA buffer (20 mM Tris-HCl, pH 7.5, 150 mM NaCl, 1 mM EDTA, 2 mM Na3VO4, 5 mM NaF, 1% Triton X-100) with Protease inhibitor (invitrogen). For IP, the protein lysates were incubated with appropriate antibodies, captured by protein G plus-agarose (Santa Cruz), and eluted by SDS loading buffer. The proteins were resolved in SDS-PAGE or Native-PAGE electrophoresis, transferred to PVDF membranes, and detected by the various primary antibodies. After incubation with appropriate secondary antibodies, membrane was visualized and analyzed by imaging system (Bio-Rad Laboratories).

### Quantitative real-time PCR (qRT-PCR) assays

Cells with appropriate treatment were collected at indicated time points. Total RNA was extracted by MolPure® Cell/Tissue Total RNA Kit (YEASEN) and quantified using a Nanodrop 2000 UV-visible spectrophotometer. cDNA was synthesized from 1 μg mRNA using the PrimeScript RT–PCR Kit (Takara) according to the manufacturer’s instructions. qRT-PCR was performed on a CFX Connect^TM^ Real-Time System (Bio-Rad Laboratories) with Hieff® qPCR SYBR Green Master Mix (YEASEN). Fold changes were calculated using the ΔΔCt method with mouse or human GAPDH as an endogenous control for mRNA expression. The sequences of primers in this assay are listed in Supplementary Table 6.

### Single cell transcriptome sequencing analysis

Single cell transcriptome data (GSE231946, subsets GSM7306384 and GSM7306386) were obtained from the Gene Expression Omnibus (GEO) database. The data came from bone marrow samples provided by two healthy people. We performed cell recognition, filtering, PCA reduction and cluster analysis on the raw data using Seurat R package. Among them, the cell filtration criteria are as follows, simultaneously meet 500 < RNA Feature <6000, 1000 < RNA Count <40,000, and mitochondrial gene proportion <20%. Then single cell clusters were visualized by t-SNE algorithm. The FindAllMarkers function was used to identify marker genes for different clusters. Marker genes were used to predict cell types. The “monocle2” R package was used to describe the cell pseudo-temporal differentiation trajectory. The single cell analysis results were visualized with the “ggplot2” R package. The “ggsci” R package was used to enrich the color of the figures.

### Gene ontology (GO) functional enrichment analysis

GO analysis is done through the database for Annotation, Visualization and Integrated Discovery (DAVID). DAVID is a comprehensive gene functional annotation database.

### Online tool for visualizing omics results

OmicStudio is an online tool that enables multiple bioinformatics data visualizations to deliver high-quality visualizations.

### Cell viability

Cell viability was detected by Cell Counting Kit 8 (NCM Biotech) following the manufacturer’s instructions. 1 × 10^5^ BM cells with indicated genotypes were plated in a well of 96-well plate, and were treated in the same condition during differentiation. Cell viability was detected at day 7. The experiment was performed in triplicate.

### LPS feeding models

8-week-old C57BL/6 female mice of indicated genotypes were fed with 160 μg LPS from *V. parvula* once by oral gavage and received 100 μg/ml LPS in drinking water for 2weeks according to previous report [74]. The serum and colon tissues were collected after euthanasia of mice for subsequent experiments.

### Inflammatory bowel disease models

8-week-old C57BL/6 female mice of indicated genotypes were fed with 5% Dextran Sulfate Sodium Salt (DSS) in drinking water for 7days. The serum and colon tissues were collected after euthanasia of mice for subsequent experiments.

### graft-versus-host (GVH) disease (GVHD) models

8-week-old WT BALB/c female mice were purchased from were purchased from the Gempharmatech Co., Ltd (Nanjing). Bone marrow (BM) cells were isolated from B6-background mice and transplanted into BALB/c mice with B6-background splenic T cells. The conditions and overall survivals of mice were recorded and analyzed.

### Immunohistochemistry (IHC) staining

Mouse colon tissues were fixed in 4% paraformaldehyde (PFA) for 24 h, embedded in paraffin, sectioned and then stained using H&E Staining Kit (Beyotime Biotechnology). For IHC staining, various tissue sections were incubated using appropriate primary antibodies and GTVisionTM III Detection System/Mo & Rb/including DAB (gene tech, Shanghai). And then cell nuclei were stained with hematoxylin (Sigma-Aldrich). The stained sections were scanned and analyzed by Leica versa 8.

### Enzyme-linked immunosorbent assay

Enzyme-linked immunosorbent assays (ELISAs) were performed according to the manufacturer’s instructions for mouse serum (R&D Systems IL-1β Quantikine ELISA, MLB00C; R&D Systems TNFα Quantikine ELISA kit, MTA00B). The plates were measured by SYNERGY2 microplate reader (Bio Tek instruments).

### LPS-induced model of sepsis in vivo

Mice of various genotypes were treated with LPS by intraperitoneal injection for LPS-induced model of sepsis. Mice were euthanized in a CO2 chamber. Blood samples were collected and serum was isolated at different time points. Then cytokines in serum were detected using ELISA kit (R&D). Survival status of mice was recorded.

### Chromatin immunoprecipitation (ChIP)-qPCR

ChIP assays were performed using ChIP Assay Kit (beyotime) following the manufacturer’s instructions. Eluted DNA were measured by qRT-PCR with input as a control. The data were analyzed by Prism software.

### Proximity ligation assays (PLA)

The PLA assays were performed using the Duolink In Situ Red Starter Mouse/Rabbit Kit (Sigma‒Aldrich) following the manufacturer’s instructions. The primary antibodies used in a PLA assay were from different sources. Images were acquired and analyzed using a confocal microscope system (Leica SP8). The PLA foci were counted and analyzed by Prism software.

### Mass spectrometric analyses

Proteomics analyses for DPEP2-associated proteins were performed as our previous report at PTM Biotech. Inc. (Hangzhou, China) [75]. Briefly, BMDM cells with indicated treatment were collected and lysed. Then, the DPEP2 and DPEP2-associated proteins were isolated using anti-DPEP2 antibodies (Proteintech), and the complexes were accumulated by Protein G beads. The protein samples were analyzed by LC-MS/MS as described above.

### CRISPR/Cas9 knockout

The sgRNA sequences were designed using the MIT online tool (http://crispr.mit.edu) and cloned into a lentiCRISPRv2 vector. Viral supernatants from HEK293T cells that were transfected by certain plasmids and packaging plasmids [pMD2. G (Addgene #12259) and psPAX2 (Addgene #12260)] were collected and concentrated. Cells were infected with concentrated viral supernatants and selected with puromycin. The colons were picked, and the knockout efficiency of clones was confirmed by Western blotting assays.

### Quantitative and statistical analysis

Unpaired Student’s *t* test and one-way ANOVA were used to analyze the data from two groups or multiple groups in this study by GraphPad Prism version 9.0 or SPSS. Statistical significance was described by *p* value and *P* values < 0.05 were considered significant. No statistical method was used to predetermine sample size. No data were excluded from the analyses. The investigators were not blinded to allocation during the experiments and outcome assessment.

## Supplementary information

Supplementary Material

table S1

table S2

table S3

table S4

table S5

table S6

aj-checklist

Original Data Files

## Data Availability

The materials and datasets used and analyzed during this study are available from the corresponding author on reasonable request.
