# Supplementary material for: The Trim32-DPEP2 axis is an inflammatory switch in macrophages during intestinal inflammation
Source: Cell Death Differ. 2025 Feb 28;32(7):1336–52. doi: 10.1038/s41418-025-01468-w (PMC12283963; doi:10.1038/s41418-025-01468-w)
Supplement: Supplementary file 1 — Supplementary Material [file 41418_2025_1468_MOESM1_ESM.docx]

**The Trim32-DPEP2 axis is an inflammatory switch** **in macrophages during** **intestinal inflammation**

Zhiyan Zhan^1,2, *^, Huisheng Liang^3, 4, *^, Zhuoqi Zhao^1, *^, Liya Pan^1^, Jing Li^1^, Yuyun Chen^5^, Zhoulonglong Xie^6^, Zhilong Yan^6^, Ying Xiang^7^, Wenxue Liu^3^, Li Hong^1^

^1^Department of Clinical Nutrition, Shanghai Children’s Medical Center, Shanghai Jiao Tong University School of Medicine, Shanghai, 200127, China

^2^Clinical Research Center, Shanghai Children’s Medical Center, Shanghai Jiao Tong University School of Medicine, Shanghai, 200127, China

^3^Department of Obstetrics and Gynecology, Zhongshan Hospital, Fudan University, Shanghai, China.

^4^Department of Gynecology, Zhongshan Hospital (Xiamen), Fudan University, Xiamen, 361000, China

^5^Fujian Children's Hospital (Fujian Branch of Shanghai Children's Medical Center), College of Clinical Medicine for Obstetrics & Gynecology and Pediatrics, Fujian Medical University, Fuzhou, China

^6^Department of Surgery, Shanghai Children’s Medical Center, Shanghai Jiao Tong University School of Medicine, Shanghai 200127, China.

^7^Department of Laboratory Medicine, Shanghai Children’s Medical Center, Shanghai Jiao Tong University School of Medicine, Shanghai, 200127, China

*These authors contributed equally to this work.

#Correspondence: Zhiyan Zhan, zhanzhiyan@sjtu.edu.cn; Li Hong, E-mail: [hongli@scmc.com.cn](mailto:hongli@scmc.com.cn); Wenxue Liu, [liu.wenxue@zs-hospital.sh.cn](mailto:liu.wenxue@zs-hospital.sh.cn); Ying Xiang, xiangying@scmc.com.cn; Zhilong Yan, dryanzhilong@163.com.

**Supplementary Figures**


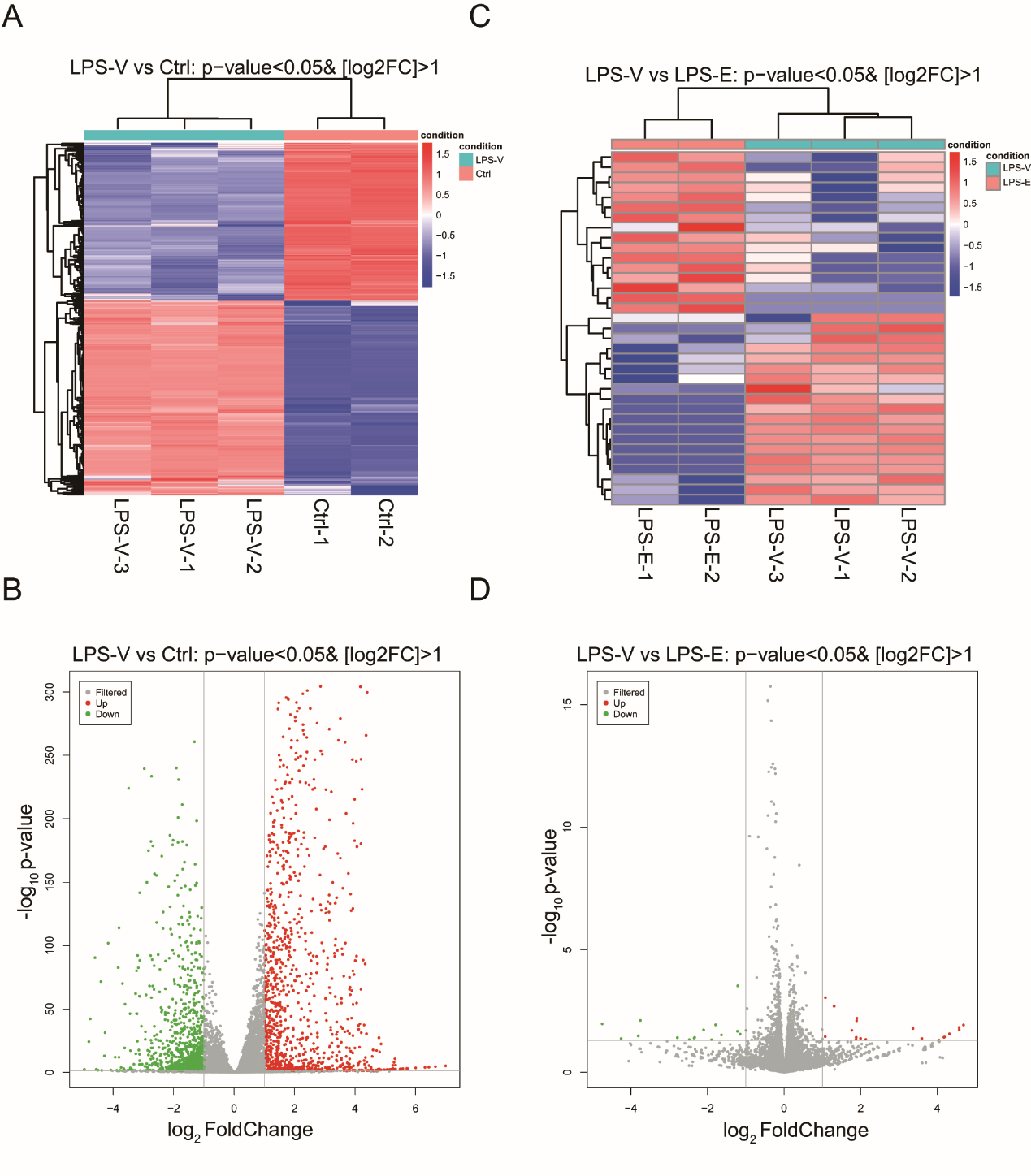


**Figure S1 The mRNA expressions of macrophages responding to different treatment.**

**A,** The heatmap of DEGs by RNA-seq in BMDMs with or without LPS-V treatment. **B,** Volcano map showing the DEGs by RNA-seq in BMDMs with or without LPS-V treatment. Red indicates highly expressed genes by LPS-V; Green indicates low expression genes by LPS-V. **C,** The heatmap of DEGs by RNA-seq in BMDMs with LPS-V or LPS-E treatment. **D,** Volcano map showing the DEGs by RNA-seq in BMDMs with or without LPS-V treatment. Red indicates highly expressed genes by LPS-V; Green indicates low expression genes by LPS-V.


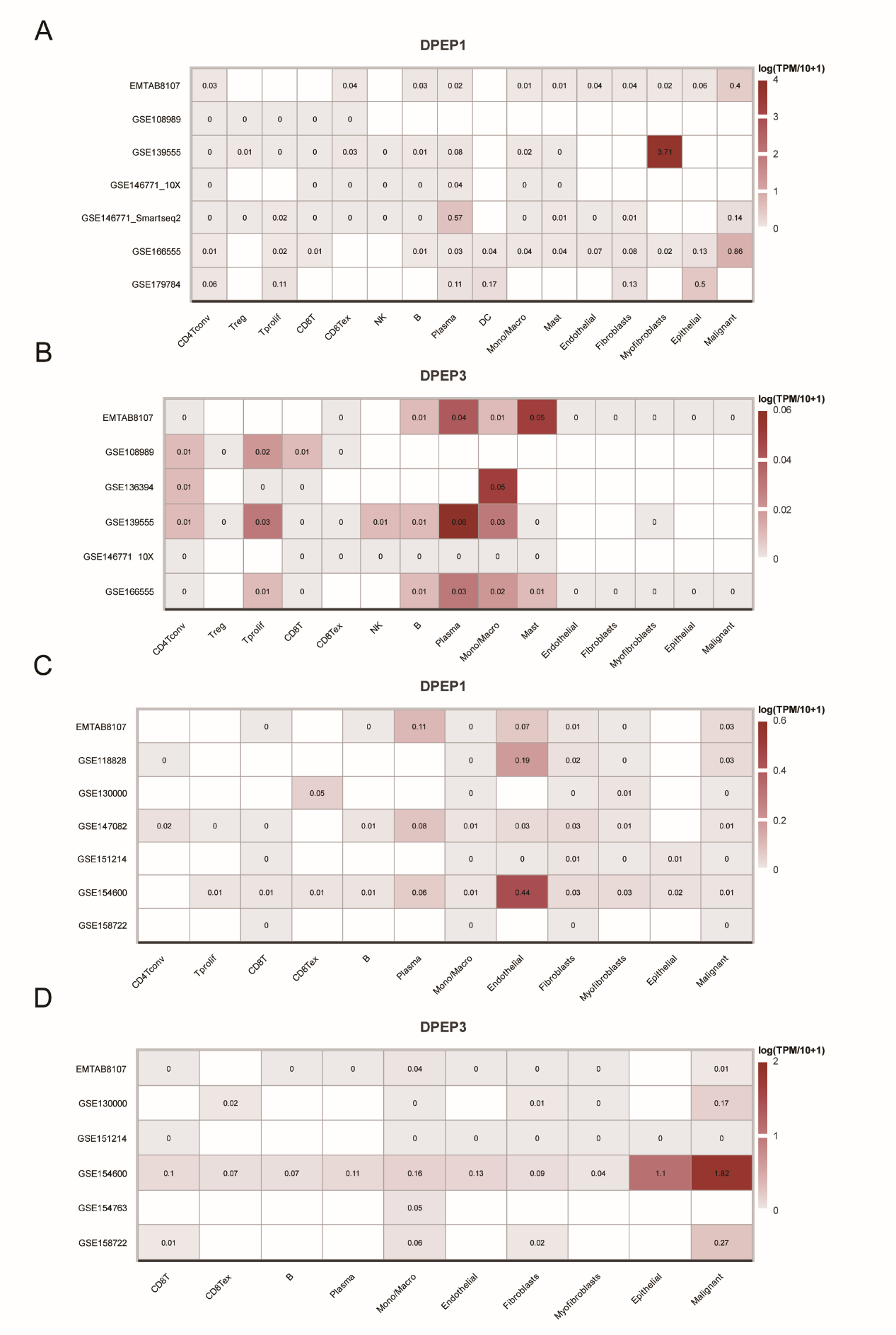


**Figure S2 The expression of DPEP1 or DPEP3 in different cell subpopulations in colorectal carcinoma and ovarian tumor tissues.**

**A,** Calculation of DPEP1 expression levels of different cell subpopulations in colorectal carcinoma tissues using the TISCH2 Dataset. **B,** Calculation of DPEP3 expression levels of different cell subpopulations in colorectal carcinoma tissues using the TISCH2 Dataset. **C,** Calculation of DPEP1 expression levels of different cell subpopulations in ovarian tumor tissues using the TISCH2 Dataset. **D,** Calculation of DPEP3 expression levels of different cell subpopulations in ovarian tumor tissues using the TISCH2 Dataset.


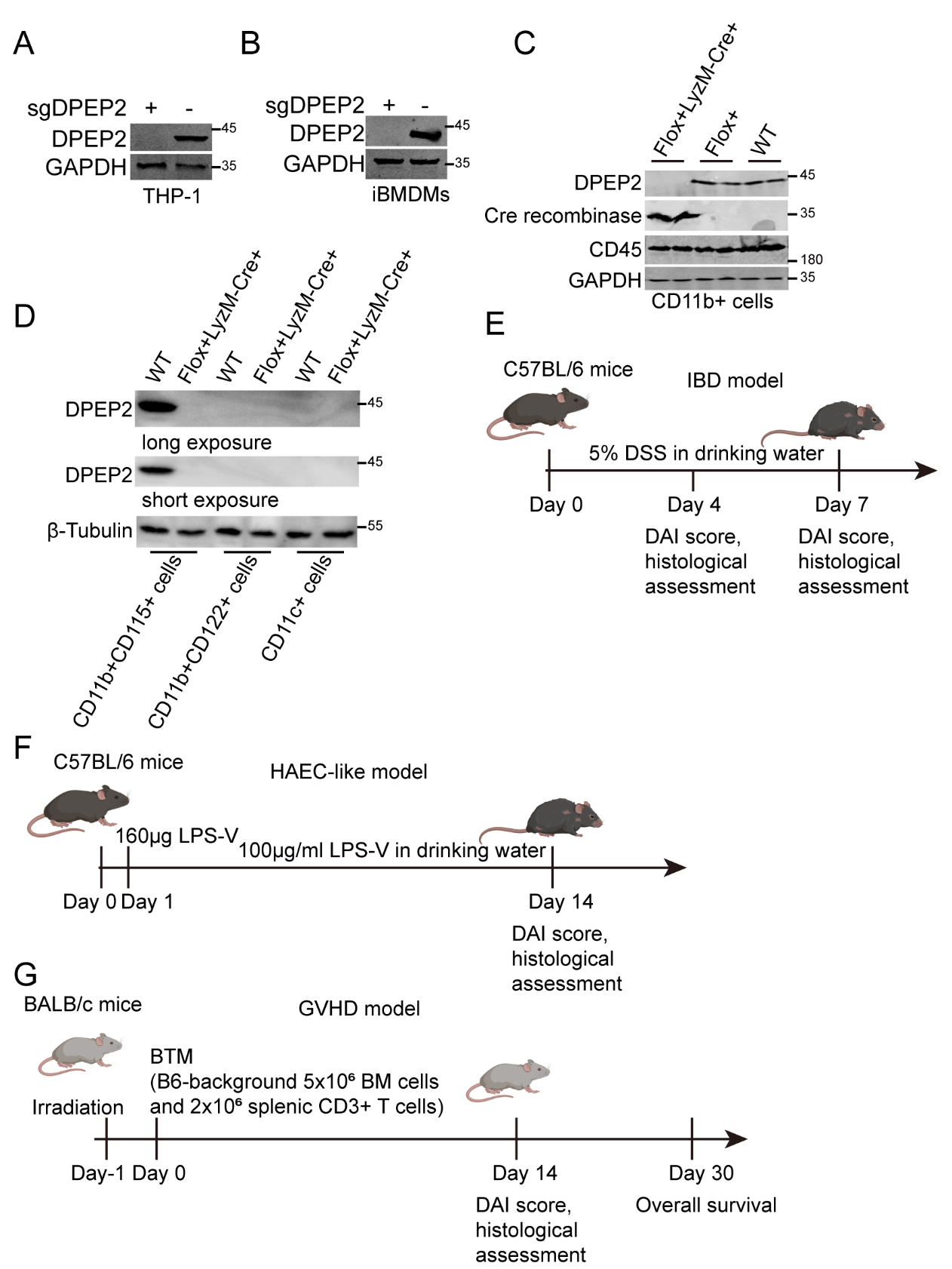


**Figure S3 Validation of cell models and description of mouse models.**

**A,** The identification of DPEP2 knockout in THP-1 cells. **B,** The identification of DPEP2 knockout in iBMDM cells. **C,** The identification of DPEP2 knockout in macrophages from the Dpep2^fl/fl^ LyzM-Cre mice. **D,** Deletion of DPEP2 protein in monocyte/macrophages from Dpep2^fl/fl^ LyzM-Cre mouse, and the DPEP2 protein was undetectable in neutrophils (CD11b+CXCR4+) from mouse or in vitro differentiated dendritic cells (CD11c+) with or without Dpep2^fl/fl^ LyzM-Cre. **E-G,** The Experimental schedules of IBD model (E), HAEC-like model (F), and GVHD model (G). The images were created by biorender.com.


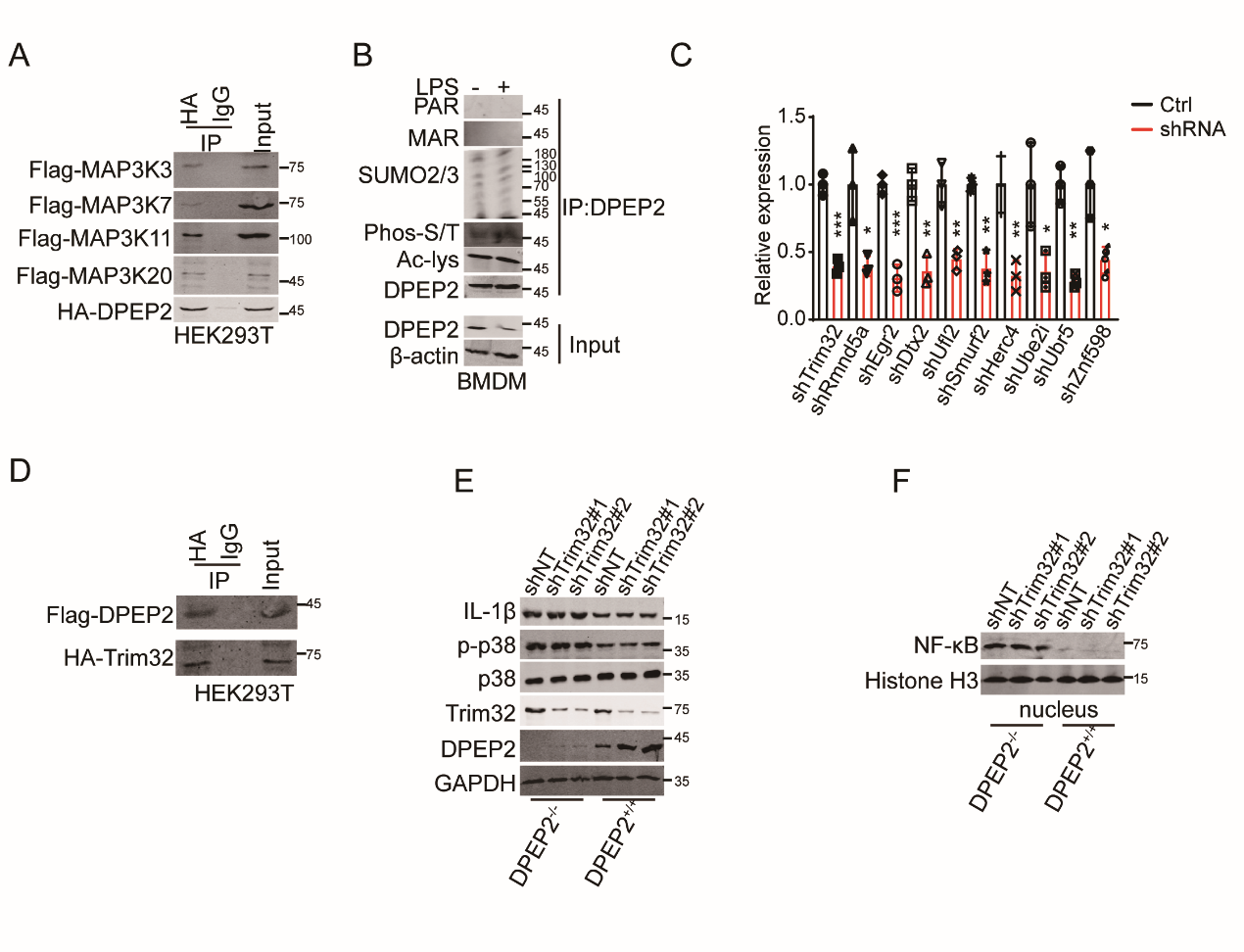


**Figure S4 DPEP2 modification by Trim32 activates the p38 and NF-κB pathways.**

**A,** The interaction of DPEP2 with MAP3K3, MAP3K7, MAP3K11, and MAP3K20 was identified in HEK293T cells using CoIP assays. **B,** The posttranslational modification of DPEP2, including PARylation (PAR), MARylation (MAR), SUMOylation, phosphorylation, and acetylation, was detected in BMDMs with LPS treatment. **D,** The interaction of DPEP2 with Trim32 was identified in HEK293T cells using CoIP assays. **E,** The effects of Trim32 knockdown on the expression of inflammatory cytokines and activation of p38 in DPEP2-KO or WT iBMDMs. **F,** The effects of Trim32 knockdown on the NF-κB activation in DPEP2-KO or WT iBMDMs. All data are expressed as mean ± SD. *P < 0.05, **P < 0.01, and ***P < 0.001.

**Table S1** The DEGs by RNA-seq in BMDMs with or without LPS-V treatment.

**Table S2** The DEGs by RNA-seq in BMDMs with LPS-E or LPS-V treatment.

**Table S3** proteomic expression in BMDMs with or without LPS-V treatment.

**Table S4** The differentially expressed proteins whose correspondent mRNA levels don’t show the same alteration.

**Table S5** DPEP2-associated proteins from BMDM cells.

**Table S6** Sequences of primers in mRNA expression analyses.
