# Supplementary material for: The Trim32-DPEP2 axis is an inflammatory switch in macrophages during intestinal inflammation
Source: Cell Death Differ. 2025 Feb 28;32(7):1336–52. doi: 10.1038/s41418-025-01468-w (PMC12283963; doi:10.1038/s41418-025-01468-w)
Supplement: Supplementary file 7 — table S6 [file 41418_2025_1468_MOESM7_ESM.docx]

**Table S6** Sequences of primers in mRNA expression analyses

|  | Sequences of forward primer | Sequences of reverse primer |
| --- | --- | --- |
| IL-1β (mouse) | TGGCAACTGTTCCTG | GGAAGCAGCCCTTCATCTTT |
| TNF-α (mouse) | GCCTCTTCTCATTCCTGCTT | TGGGAACTTCTCATCCCTTTG |
| IL-6 (mouse) | CTGCAAGAGACTTCCATCCAG | AGTGGTATAGACAGGTCTGTTGG |
| DPEP1(mouse) | GCACAACGACTTGCCTTGG | ATGCGGTGTATCACATCCATC |
| DPEP2(mouse) | CACAGCCTCTAATATGGACCCA | GCTGGAAATGTCGGGCTTG |
| DPEP3(mouse) | CTGCTGGGGGTGTTACTTCTA | CGTGGCCGTGTCATTAGGAA |
| GAPDH (mouse) | TCCCACTCTTCCACCTTCGA | AGTTGGGATAGGGCCTCTCTT |
| DPEP2 (human) | CAGCCTGTAACCTGTGCCTAC | GCTGGGACTACTGAGTGTGG |
| GAPDH (human) | AGCCGAGCCACATCGCT | GCAACAATATCCACTTTAC |
