# Supplementary figures and images for: The Trim32-DPEP2 axis is an inflammatory switch in macrophages during intestinal inflammation
Source: Cell Death Differ. 2025 Feb 28;32(7):1336–52. doi: 10.1038/s41418-025-01468-w (PMC12283963; doi:10.1038/s41418-025-01468-w)

Figure1

F

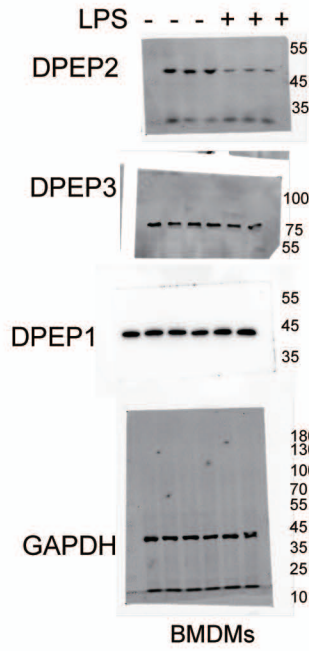

G

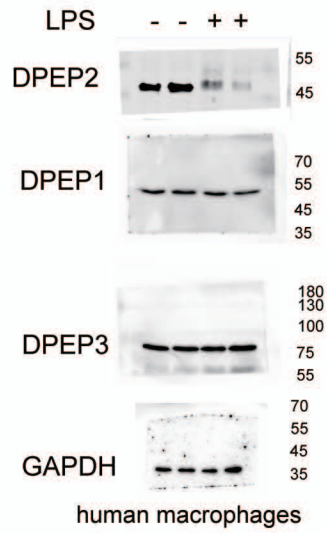

Figure2

G

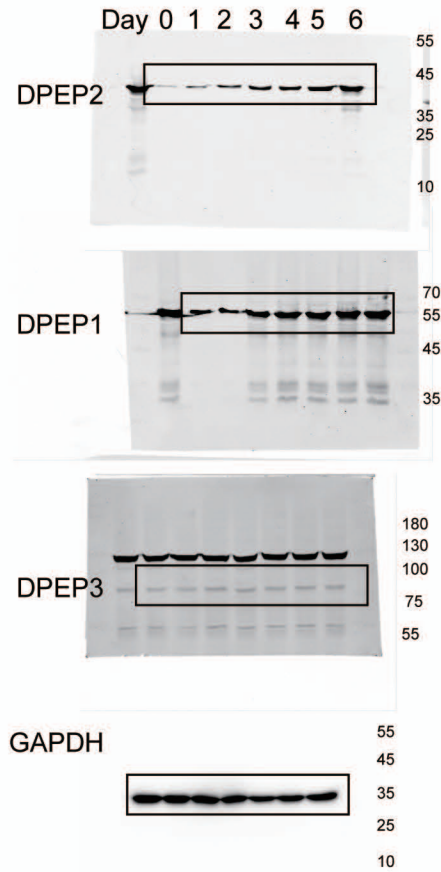

I

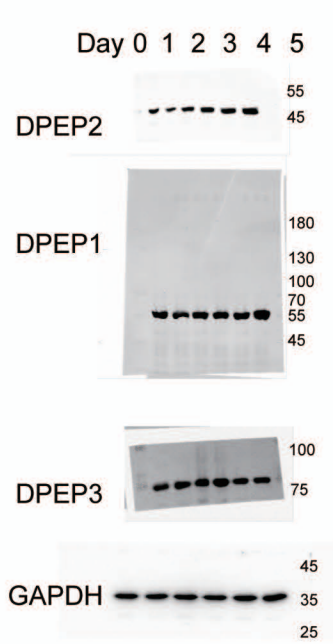

K

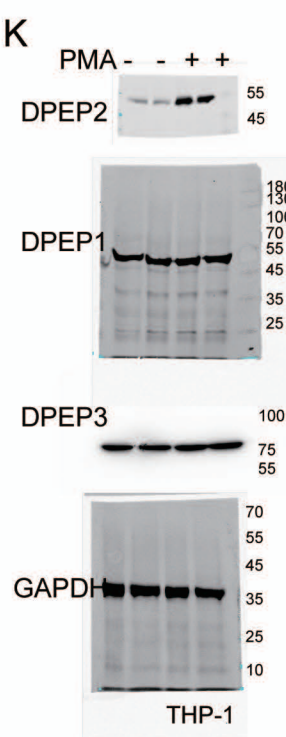

J

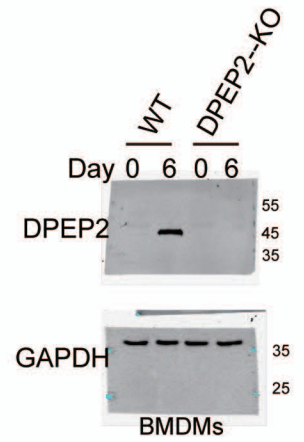

Figure3  
G

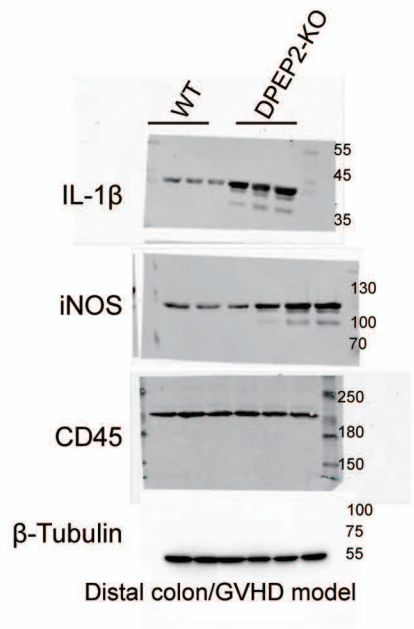

Figure 4

C

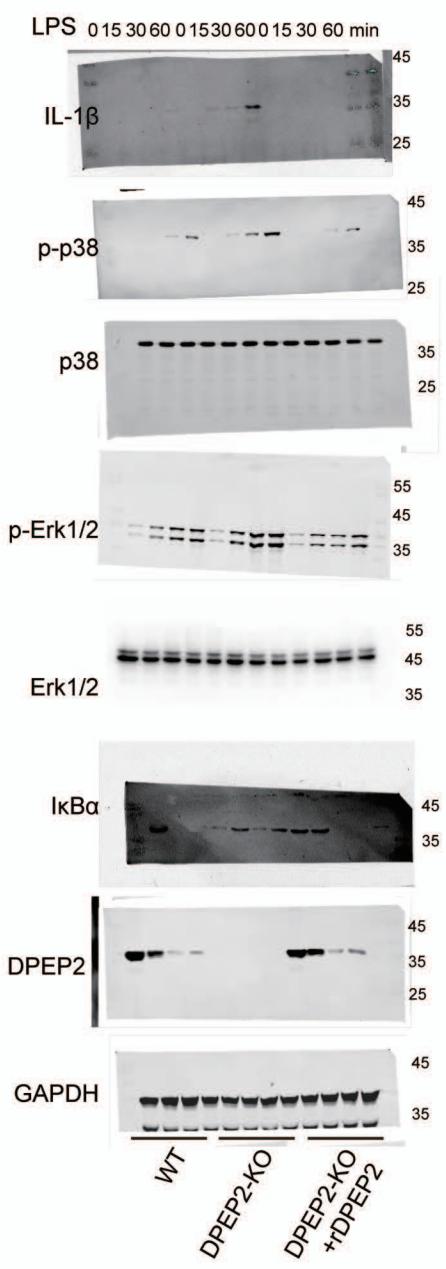

D

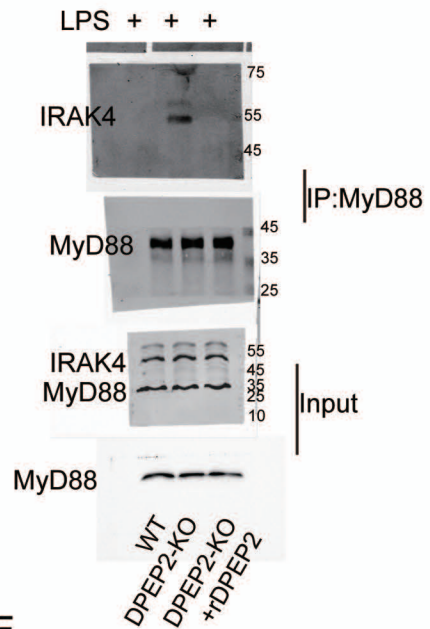

E

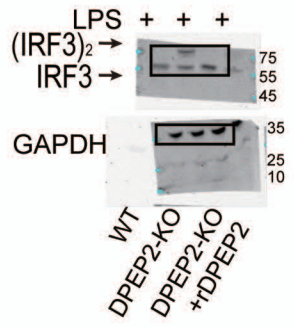

F

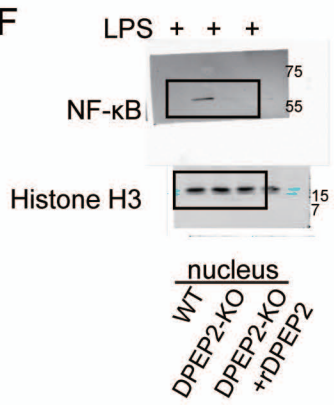

Figure 5

C

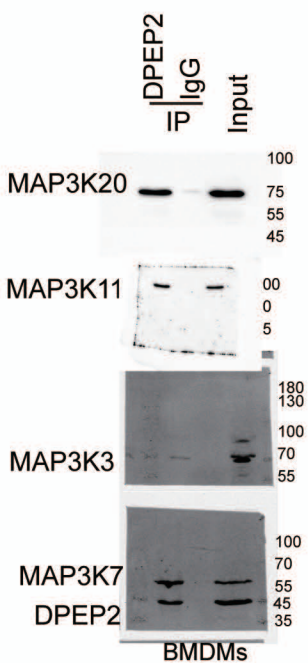

D

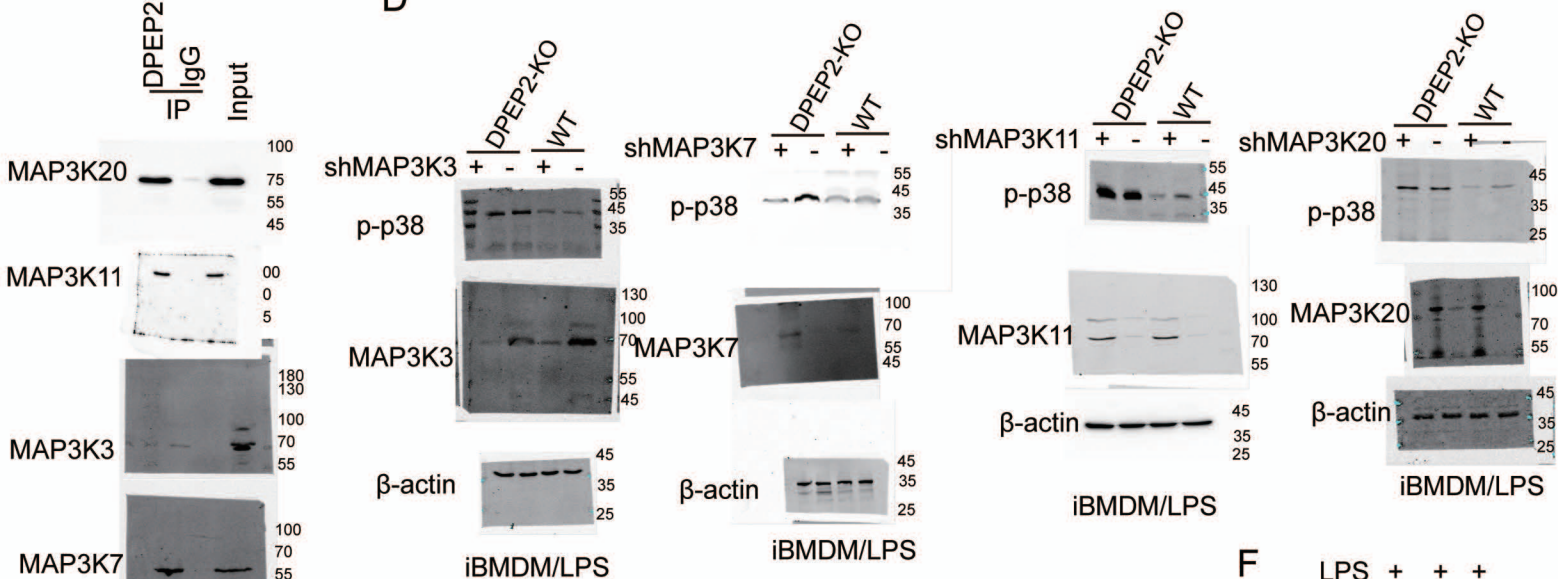

F

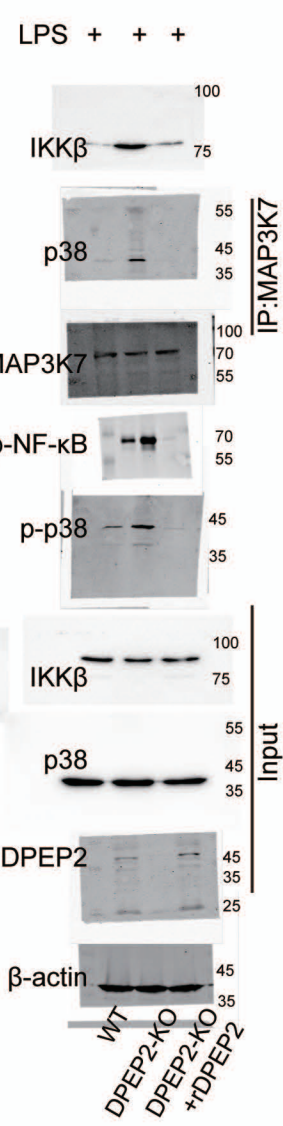

E

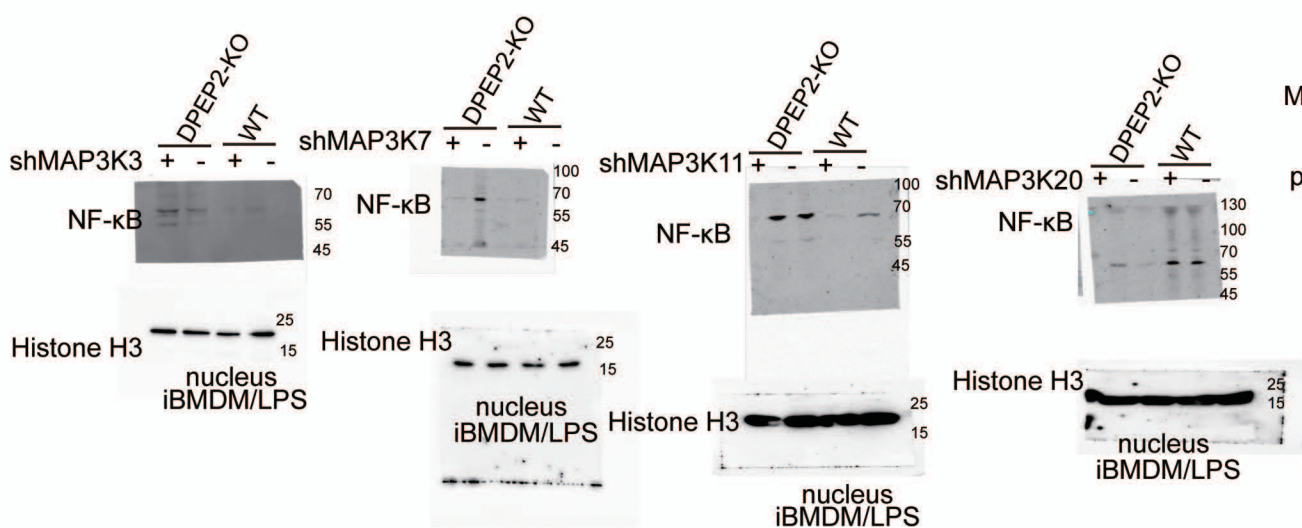

A

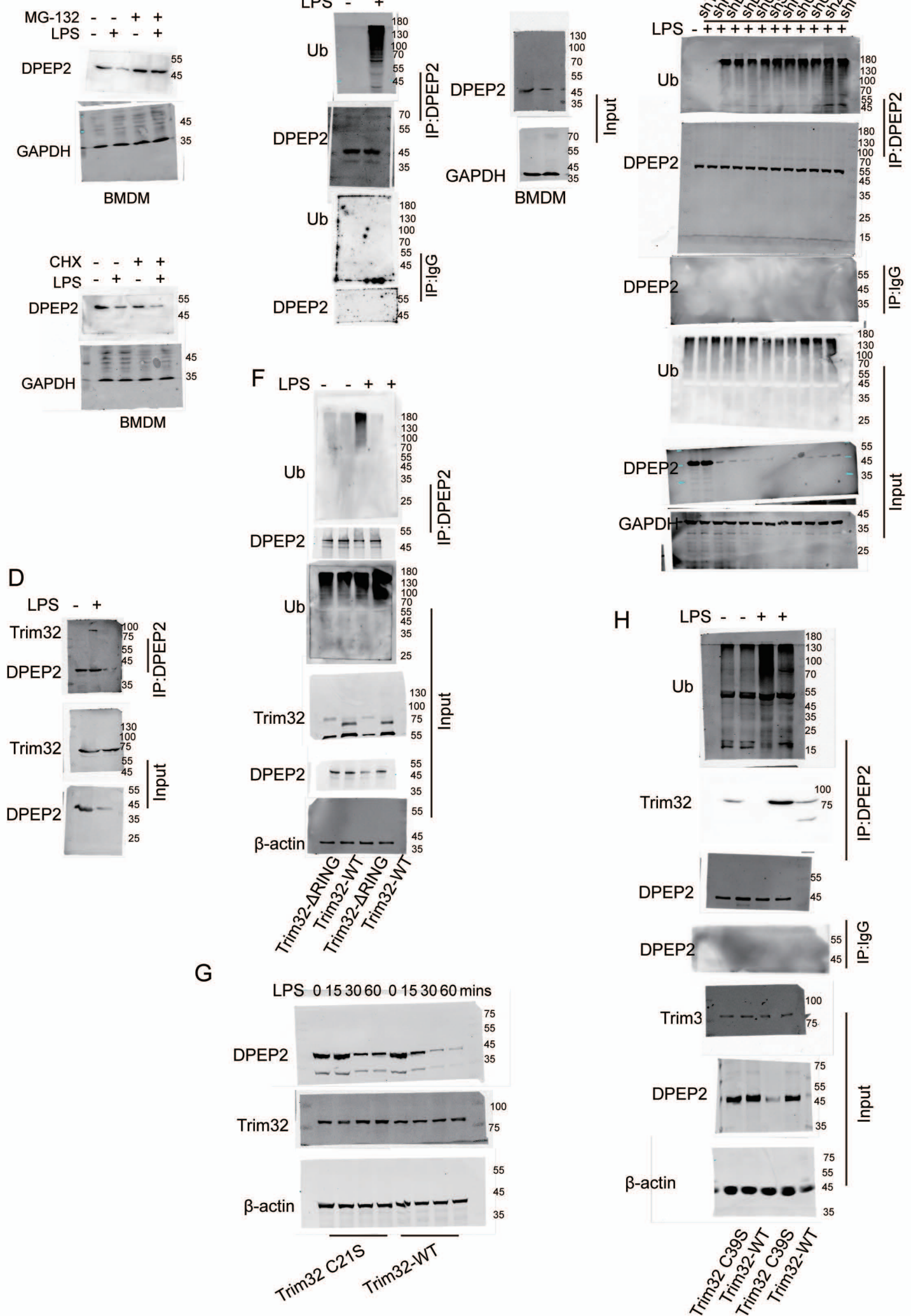

Figure 7

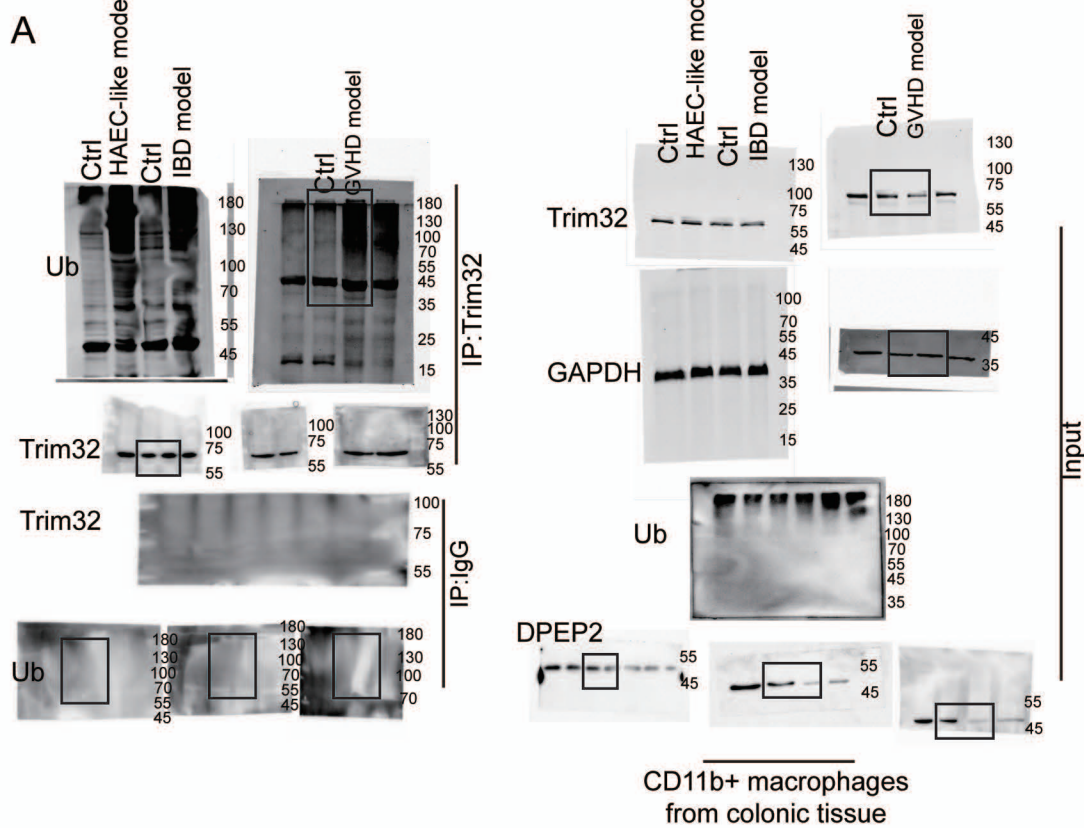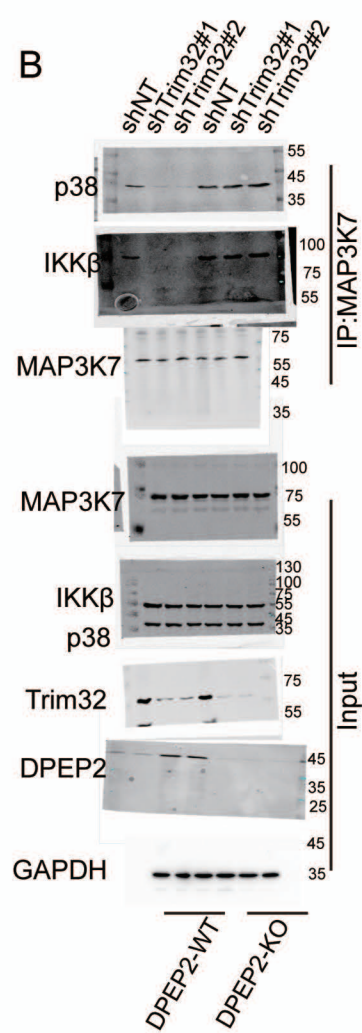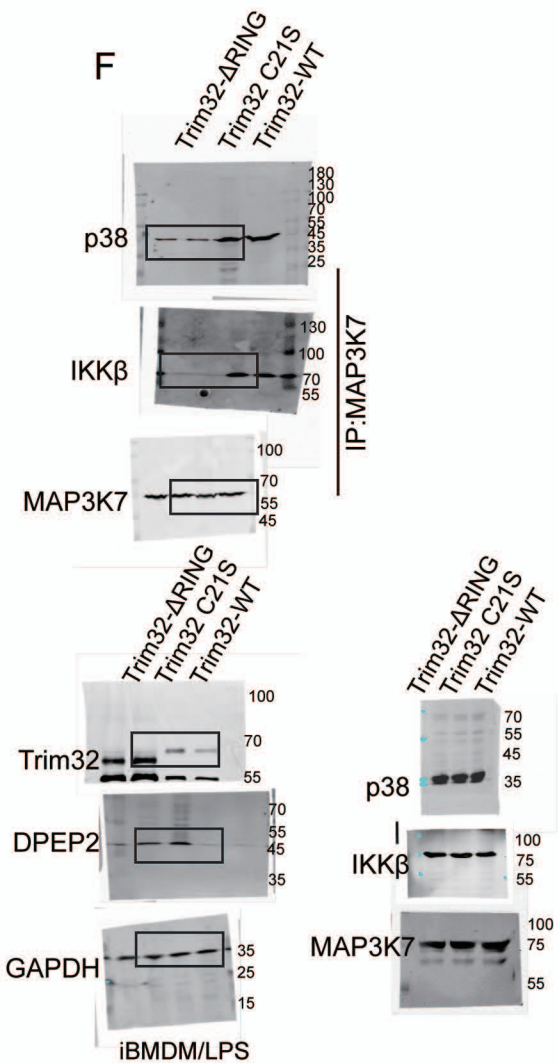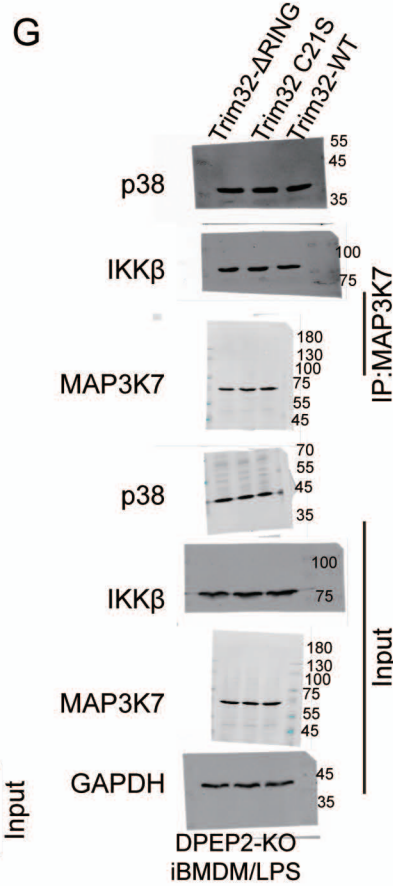

Figure 8

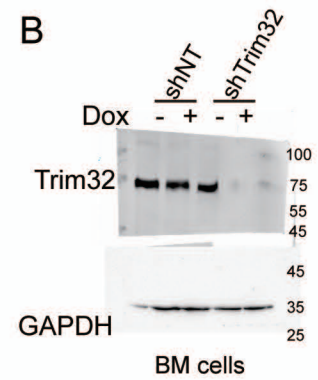

Figure S3

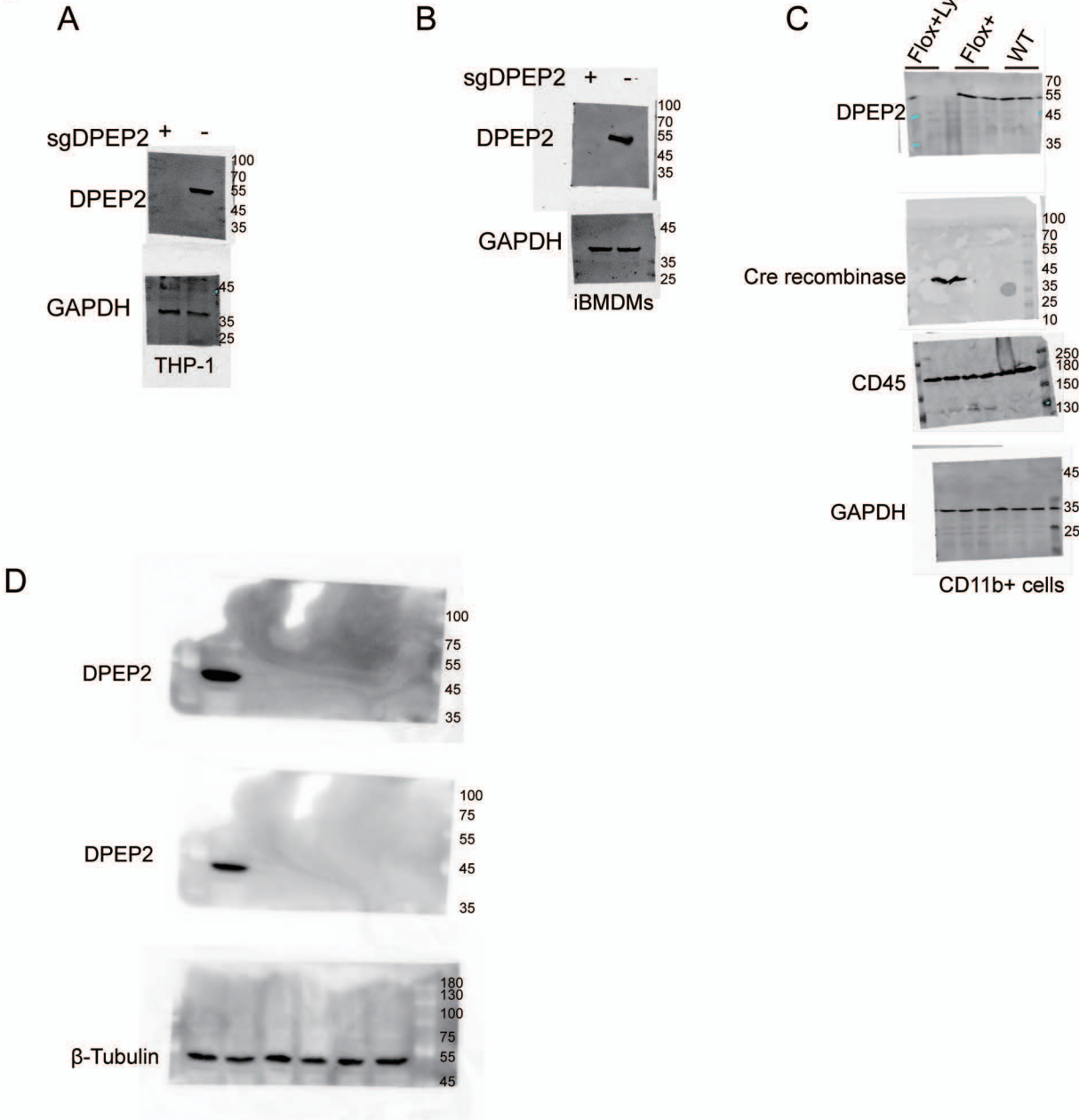

Figure S4

A

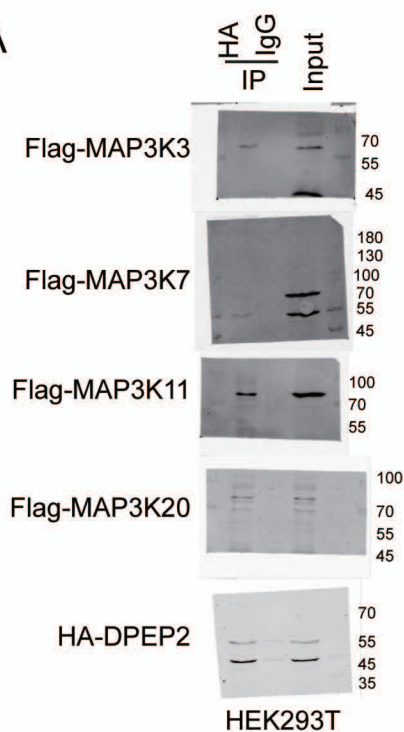

B

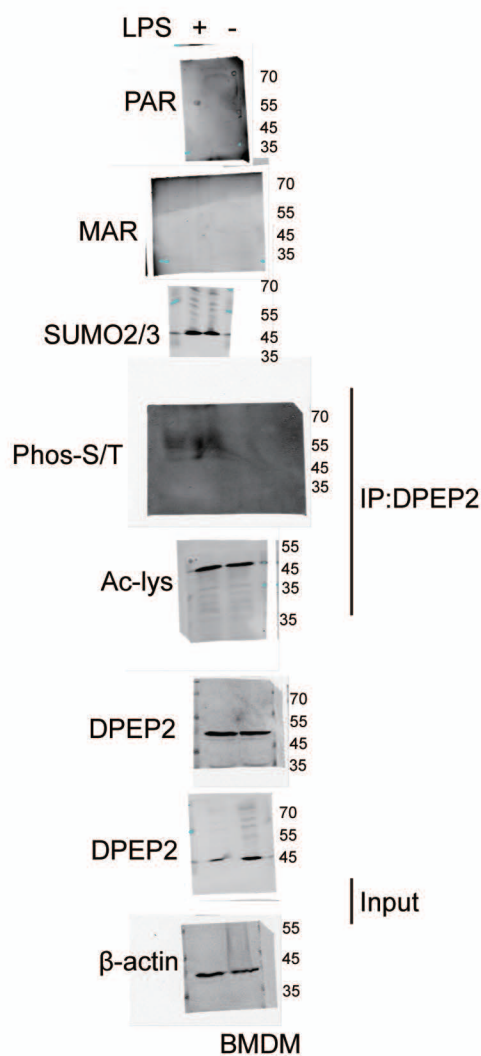

D

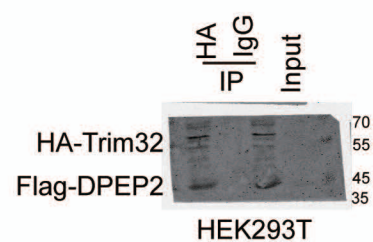

E

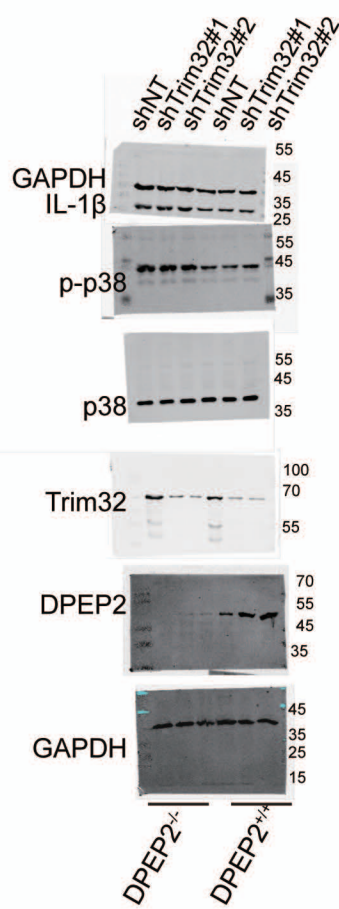

F

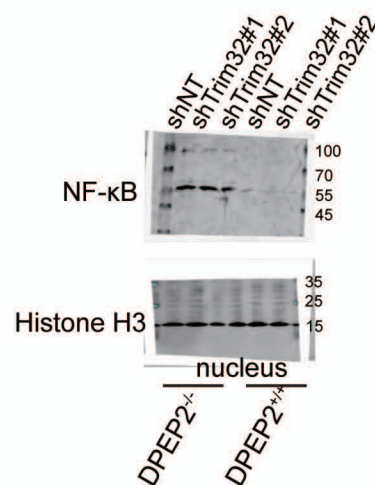

Supplement: Supplementary file 9 — Original Data Files [file 41418_2025_1468_MOESM9_ESM.pdf]
